# APE1 condensation in nucleoli of non-cancer cells depends on rRNA transcription and forming G-quadruplex RNA structures

**DOI:** 10.1093/nar/gkaf168

**Published:** 2025-03-18

**Authors:** Giuseppe Dall’Agnese, Nancy M Hannett, Kalon J Overholt, Jesse M Platt, Jonathan E Henninger, Asier Marcos-Vidal, Zahraa Othman, Gilmar Salgado, Giulia Antoniali, Gianluca Tell

**Affiliations:** Laboratory of Molecular Biology and DNA Repair, Department of Medicine, University of Udine, 33100 Udine, Italy; Whitehead Institute for Biomedical Research, 455 Main Street, Cambridge, MA 02142, United States; Whitehead Institute for Biomedical Research, 455 Main Street, Cambridge, MA 02142, United States; Whitehead Institute for Biomedical Research, 455 Main Street, Cambridge, MA 02142, United States; Department of Biological Engineering, Massachusetts Institute of Technology, Cambridge, MA 02139, United States; Whitehead Institute for Biomedical Research, 455 Main Street, Cambridge, MA 02142, United States; Division of Gastroenterology, Department of Medicine, Massachusetts General Hospital, Boston, MA 02114, United States; Whitehead Institute for Biomedical Research, 455 Main Street, Cambridge, MA 02142, United States; W.M. Keck Imaging Facility, Whitehead Institute for Biomedical Research, Cambridge, MA 02142, United States; ARNA Laboratory—Inserm U1212—CNRS UMR 5320, Bordeaux Biologie Santé—Université de Bordeaux, 146 Rue Léo Saignant, 33076 Bordeaux, France; ARNA Laboratory—Inserm U1212—CNRS UMR 5320, Bordeaux Biologie Santé—Université de Bordeaux, 146 Rue Léo Saignant, 33076 Bordeaux, France; Laboratory of Molecular Biology and DNA Repair, Department of Medicine, University of Udine, 33100 Udine, Italy; Laboratory of Molecular Biology and DNA Repair, Department of Medicine, University of Udine, 33100 Udine, Italy

## Abstract

APE1 [apurinic/apyrimidinic (AP) endodeoxyribonuclease 1] is the main endonuclease of the base excision repair pathway acting on abasic (AP) sites in DNA. APE1 is an abundant nuclear protein, and improper expression or localization of this factor could lead to the accumulation of toxic DNA intermediates. Altered APE1 subcellular distribution and expression are associated with cancer development, suggesting the importance of a fine-tuning mechanism for APE1 activities. Recent works highlighted the presence of APE1 within nucleoli of cancer cells and the ability of APE1 to form biomolecular condensate. However, whether secondary structures of ribosomal RNA (rRNA) influence the nucleolar localization of APE1 remains poorly understood. Since protein overexpression can result in artificial nucleolar accumulation, it is imperative to have appropriate cellular models to study APE1 trafficking under physiological conditions. To address this issue, we generated a murine embryonic stem cell line expressing endogenous fluorescent-tagged APE1. Live-cell imaging demonstrates that APE1 nucleolar accumulation requires active rRNA transcription and is modulated by different genotoxicants. *In vitro* experiments showed that APE1 condensate formation depends on RNA-forming G-quadruplex structures and relies on critical lysine residues. This study sheds light on the mechanisms underlying APE1 trafficking to the nucleolus and the formation of RNA-dependent APE1 nucleolar condensates.

## Graphical Abstract

**Graphical Abstract:**
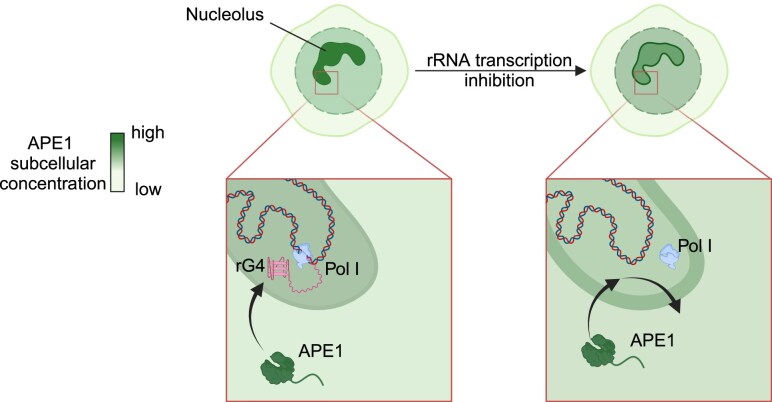

## Introduction

Damage to DNA is a threat to the cell, and different DNA damage response (DDR) pathways have been acquired during evolution to cope with insults to DNA. These pathways involve the coordinated action of different proteins and enzymes towards several toxic intermediates that should be promptly and efficiently repaired in order to preserve genomic stability and prevent the activation of the death signalling cascades [1]. APE1 [apurinic/apyrimidinic (AP) endodeoxyribonuclease 1] is an enzyme discovered for its central role as the main endonuclease acting on the base excision repair (BER) pathway on abasic (AP) sites generated spontaneously or by the action of several glycosylases [2, 3]. APE1 is also known for its additional, non-canonical functions such as redox-regulated activity on different transcription factors [4], microRNA processing [5, 6], and RNA quality control [7, 8]. APE1 is an abundant protein within human cells, with concentrations ranging from ∼0.2 to 10 μM [9], which is up to 100-fold higher than the downstream enzymes in the BER pathway [10]. Improper quantitative expression of the protein could therefore generate the accumulation of toxic DNA intermediates, mainly single-strand breaks (SSBs). The APE1 C-terminus, responsible for the endonuclease activity, through which it generates SSBs upon recognition of the abasic sites, is highly conserved during evolution; meanwhile, its N-terminus (comprising residues 1–33), responsible for the non-canonical functions of the protein, seems to be recently acquired during phylogenesis [11]. It is widely known that APE1 is mainly present in the nucleus with a described nucleolar enrichment [12] due to its interaction with NPM1 [7, 13]. Several pathologies, mostly cancers, are associated with either altered APE1 subcellular localization [14–16], expression levels (mostly overexpression) [17–19], or hyperacetylation [20, 21], suggesting the importance of a fine-tuning mechanism for APE1 nuclear-associated processes.

Our lab has previously shown that APE1 endonuclease activity participates in ribosomal RNA (rRNA) quality control and nucleolar enrichment through the interaction with NPM1 [16, 22–26]. Biochemical purification of nucleoli showed that APE1 nucleolar enrichment, along with other BER-associated proteins, is highly regulated by NPM1; either NPM1 knockdown or its subcellular re-localization upon cisplatin (CDDP) treatment leads to APE1 nucleolar depletion [25]. We previously characterized several APE1-acetylated mutants by using cellular reconstitution assays: HeLa cell clones are able to express a specific, inducible APE1-siRNA to knock down the endogenous protein (protein levels lower than 10% of the control) and re-express ectopic FLAG-tagged forms through small interfering RNA (siRNA)-resistant complementary DNA-expressing plasmids. With this cellular model, we discovered that acetylation of lysine K^27^/K^31^/K^32^/K^35^ occurs upon genotoxic stimuli, and in addition to being relevant for APE1–NPM1 interaction, it plays essential roles in both APE1 DDR-associated enzymatic activity [22] and nucleic acid interactions [10, 24]. Several labs have investigated the role of acetylation on lysine at the APE1 N-terminal region, comprising the first 33 N-terminal amino acids being part of an intrinsically disordered region (IDR) of the protein, demonstrating the interconnection between various regulatory factors [27]. Additionally, acetylation of APE1 N-terminal lysines seems to play a newly discovered role in the binding and stabilization of DNA G-quadruplex (G4) telomeric sequences [28–31].

Recent evidence suggests that ribosomal DNA (rDNA) could form G4 [32] structures, which indeed are also observed forming in rRNA, leading to RNA-forming G-quadruplex (rG4) [33]. rG4s are known to play different roles in cellular biology, ranging from telomere protection, regulation of transcription, inhibition of translation, and replication-dependent genome instability [34]. Interestingly, rG4s seem to play a role also in condensate formation that controls nuclear and nucleoli assembly [35, 36]. Nucleoli play a fundamental role as (i) a ‘stress sensor’ by regulating the p53 signalling pathway [37], (ii) a source of transcription of different RNA molecules beside rRNA [38], and (iii) a hub for maintenance of the genome stability since they are able to coordinate and accumulate over 150 different DDR-associated proteins [39]. All of those non-canonical nucleolar-associated mechanisms have been summarized in [40]. Considering that rRNA is predicted to fold into several rG4 structures, it might be possible that many nucleolar functions can be modulated by dynamically regulated rG4 structures. Interestingly, while APE1’s ability to bind G4 structures in DNA has been previously reported, no evidence for a similar activity on rG4 has yet been shown and could represent a novel hypothesis for mechanistically explaining APE1 nucleolar enrichment. Nucleolar assembly is known to be formed through a phase separation process involving condensate formation. Condensates, defined as dynamic organelles not enclosed within membranes, are subcellular compartments mostly formed by protein and nucleic acids [41], in which post-translational modifications (PTMs) are known to play important roles (e.g. acetylation is known to disrupt condensates) [42, 43]. Interestingly, condensates are emerging as important players in modulating DDR [1]. Remarkably, common aspects of all those DDR proteins able to form or to be compartmentalized into DDR condensates include the presence of an IDR and the ability to bind to RNA, two characteristics that help proteins undergo phase separation [44–48].

Recently, Li *et al.*, using overexpression cancer cell models, showed that APE1 can undergo phase separation, promoting ATR-Chk1 condensate formation in the nucleolus [49]. Ataxia-Telangiectasia Mutated and Rad3 Related (ATR) plays an important role in regulating cell cycle checkpoints as well as the homologous repair (HR) pathway. With the consideration that a recent study demonstrated that APE1 overexpression is involved in the dysregulation of the HR pathway [19], the generation of a better model, with physiological expression of APE1, is needed to investigate APE1’s ability to undergo phase separation in living cells under normal conditions. Additionally, this model could be useful to investigate the potential role that rG4 plays in promoting APE1 condensate formation.

Until now, a major limitation of all the studies concerning APE1 subcellular distribution and functions is that they have been conducted using overexpression cell models and primarily immunofluorescence analyses utilizing fixation methods that could cause artefacts of nucleolar accumulation of some dynamic, weakly binding nuclear proteins, such as HMGB1 and histone H1, as recently suggested [50]. Considering the mentioned issues, there is an urgent need for additional cell models to characterize the functions of proteins under more physiological conditions and for live imaging cell analyses.

In this work, we generated and deeply characterized what, to our knowledge, is the first non-tumour cell line [i.e. murine embryonic stem cell (mESC)] expressing an endogenous fluorescently tagged APE1. Using this mESC model, we were able to follow, in live-cell imaging, the dynamics of APE1 subcellular trafficking under basal conditions and different genotoxic treatments, showing that nucleolar enrichment of the protein was dependent on active rRNA transcription and could be modulated by CDDP treatment. Moreover, using purified recombinant proteins, we methodically assessed and quantified the influence of DNA and RNA on APE1 droplet formation *in vitro*, showing that rG4s present in rRNA genes stimulate APE1 condensate formation. Findings from this study provide the basis for understanding the molecular mechanisms responsible for APE1 nucleolar distribution in normal cells.

## Materials and methods

### Cell lines and culture conditions

V6.5 mESCs were taken from a male individual derived from a cross of C57BL/6(F) × 129/sv(M) and were kindly provided by the Jaenisch laboratory of the Whitehead Institute.

mESCs were cultured at 37°C, 5% CO_2_ in a humidified incubator on tissue-culture plates covered by 0.2% gelatin (Sigma, G1890) in 2i medium with LIF, made by 960 ml Dulbecco’s modified Eagle’s medium (DMEM)/F12 (Life Technologies, 11320082), 5 ml N2 supplement (Life Technologies, 17502048; stock 100×), 10 ml B27 supplement (Life Technologies, 17504044; stock 50×), 5 ml additional l-glutamine (Gibco, 25030-081; stock 200 mM), 10 ml Minimum Essential Medium (MEM) non-essential amino acids (Gibco, 11140076; stock 100×), 10 ml penicillin–streptomycin (Life Technologies, 15140163; stock 10^4^ U/ml), 333 μl bovine serum albumin (BSA) fraction V (Gibco, 15260037; stock 7.50%), 7 μl β-mercaptoethanol (Sigma, M6250; stock 14.3 M), 100 μl leukemia inhibitory factor (LIF - Chemico, ESG1107; stock 10^7^ U/ml), 100 μl PD0325901 (Stemgent, 04-0006-10; stock 10 mM), and 300 μl CHIR99021 (Stemgent, 04-0004-10; stock 10 mM).

When cells were passaged, TrypLE inhibition occurred using stem cell media (SCM) made by 500 ml DMEM KO (Gibco, 10829-018), MEM non-essential amino acids (Gibco, 11140076; stock 100×), 5 ml penicillin–streptomycin (Life Technologies, 15140163; stock 10^4^ U/ml), 5 ml l-glutamine (Gibco, 25030-081; stock 100×), 4 μl β-mercaptoethanol (Sigma, M6250; stock 14.3 M), 50 μl LIF (Chemico, ESG1107; stock 10^7^ U/ml), and 75 ml of fetal bovine serum (Sigma, F4135). Cells were then pelleted by centrifugation at 1000 rpm for 3 min and resuspended in 2i medium containing LIF.

Freezing of cells was performed in SCM, supplemented with a 1:1 ratio of 2× freezing media: 60 ml DMEM (Gibco, 11965-052), 20 ml dimethyl sulfoxide (Sigma, D2650), and 20 ml fetal bovine serum (Sigma, F4135).

Human embryonic stem cell (hESC) H1, purchased from WiCell, was cultured and fixed in 4% paraformaldehyde (PFA) on coverslips by Ido Sagi.

### Generation of plasmids for endogenous tagging of APE1 in mESC

Repair templates for tagging of APE1 at endogenous loci were generated on a pUC19 backbone with left and right homology arms derived from genomic DNA; primers are listed in Supplementary Table S1. Polymerase chain reactions (PCRs) were assembled using Phusion Flash High-Fidelity PCR Master Mix (Life Technologies, F548S) according to manufacturer’s instructions. PCR products were run in agarose gel, purified, and assembled using the Gibson Assembly Cloning Kit (NEB, E5510S) according to manufacturer’s instructions. gRNA–Cas9 plasmids were generated upon cleavage with the BbsI restriction enzyme (NEB, R3539M) of the px330 plasmid following NEB instructions; the cleaved plasmid was run on gel and purified. Complementary guide RNA (gRNAs - listed in Supplementary Table S1) were ordered. The annealed gRNAs were ligated to the cleaved px330 plasmid using T4 DNA ligase (NEB, M0202T) and specific buffer (NEB, B0202S) following manufacturer’s instructions. All primers and gRNA were checked for single occupancy in the genome using the BLAT tool available on the UCSC genome browser. All primers were purchased from Eton; plasmid sequences were either checked by Sanger sequencing (Eton), for gRNA–Cas9 containing plasmids, or by Nanopore sequencing (Plasmidsaurus), for repair templates. APE1 was tagged with mEGFP, HA, and FLAG tags, allowing for protein purification or immunocapturing.

### Tagging of APE1-mEGFP endogenous cell lines

The following procedure was applied for both N- and C-terminus tagging strategies. First, 1 × 10^6^ cells were plated with SCM, serum-deprived, in one well of a six-well plate covered in 0.2% gelatin and reverse transfected with 1.6 μg of the plasmid containing the repair template, 0.4 μg of each plasmid containing gRNA1 and gRNA2 using Lipofectamine 3000 (Life Technologies, L3000008) following manufacturer’s instructions. Culturing medium was changed the day after transfection with the 2i medium with LIF. After 2 days, cells were sorted based on mCherry^+^ (tag encoded with Cas9) to enrich the population for transfected cells. mCherry^+^ cells were expanded for 7 days before the second sorting based on the mEGFP signal. All mEGFP^+^ cells were expanded, and at a later stage, dilution plating on a 10-cm dish was performed to allow single colony picking. Clones were picked and expanded into a 96-well plate; upon confluence, three replicates of the 96-well plate were made to (i) freeze down cells and (ii) access the zygosity condition of the clones (primers used are listed in Supplementary Table S1C). Selected clones were expanded and fully characterized as described in the ‘Result’ section.

### Western blot

Cells were harvested and resuspended with lysis buffer [50 mM Tris–HCl (pH 7.5), 150 mM NaCl, 1 mM ethylenediaminetetraacetic acid (EDTA; pH 8.0), 1% Triton X-100] supplemented with 1 mM protease inhibitor cocktail and 0.5 mM phenylmethylsulfonyl fluoride. After 20 min of incubation on ice, lysates were centrifuged at 15 000 rpm to remove debris, and whole cell extract was quantified using the BCA Protein Assay Kit (Life Technologies, 23250) following manufacturer’s instructions. Cell extracts were kept at −80°C. Equal amounts of protein were separated on 10% Bis–Tris gels (Bio-Rad Laboratories, 3450112) and transferred to a 0.45-μm PVDF membrane (Sigma, IPVH00010). Upon transfer, membranes were blocked in 5% non-fat milk (LabScientific, M0842) in Tris-buffered saline with Tween 20 (TBST) - [2% Tris–HCl (pH 8.0), 1.3% 5 M NaCl, 0.05% Tween-20] for 30 min at room temperature (RT) with shaking. Membranes were then incubated with primary antibody anti-APE1 (Novus Biologicals, NB100-101) and anti-βActin (Sigma, A5441) in 5% non-fat milk in TBST. Secondary antibodies—goat anti-rabbit IRDye-800CW (LI-COR, 926-32211) and goat anti-mouse IRDye-680RD (LI-COR, 926-68070)—in 5% non-fat milk in TBST were used to detect protein signal, assessed using Odyssey CLx LI-COR, and quantified with Image Studio.

### Cell cycle analysis

Total 2 × 10^6^ cells were harvested and resuspended in 10 ml of −20°C-cold 90% ethanol in phosphate-buffered saline (PBS). Cells were then kept at −20°C for at least 1 h. When ready, cells were centrifuged to remove ethanol and rehydrated with 10 ml of PBS. Upon rehydration, cells were collected by centrifugation and resuspended in 2 ml of PBS containing 10 μg/ml of RNaseA (Invitrogen, 8003088) and 20 μg/ml PI (Life Technologies, P3566) for 30 min and then analysed with the cell sorter. A minimum of 11 000 cells were measured for each experimental condition. Analyses of the results were performed using FlowJo.

### MTS viability assay

Total 2 × 10^4^ cells were plated in a 96-well plate covered in 0.2% gelatin with 2i medium with LIF. The following day, cells were treated with different concentrations of CDDP (Fisher Scientific, 50148565) for 24 h or methyl methanesulfonate (MMS; Millipore, 129925) for 8 h. One hour before the end of the treatment, CellTiter-Glo (Promega, G9241) was added to the media, following the manufacturer’s instructions, and incubated for 1 h prior to recording the absorbance at 490 nm with Tecan Safire 2. Each recorded absorbance value was standardized with the absorbance value of the wells that contained medium only. The experiment was performed in four independent experiments, each time with a technical triplicate.

A similar experiment was conducted by treating cells with 50 μM CDDP or 0.5 mM MMS for 6 h to evaluate the cell metabolic activity of the cells.

### Immunofluorescence and image analysis

Here, 1 × 10^5^ cells were plated in a glass bottom 24-well plate (Mattek, P24G-1.5-13-F) pre-treated with 5 μg/ml of poly-L-ornithine (Sigma, P4957) at 37°C for at least 1 h followed by 5 μg/ml of laminin (Corning, 354232) overnight at 37°C. After overnight plating, cells were treated with different doses of CDDP for 24 h or with MMS for 8 h and fixed with 4% PFA (VWR, AA47377) for 20 min and stored in PBS at 4°C. Cells were permeabilized for 5 min with 0.5% Triton X-100 (Sigma, X100) in PBS and blocked with 5% IgG-free BSA (VWR, 102643-516) in PBS. Anti-pS139 γH2AX (Sigma, 05-636) in 5% BSA followed by Alexa Fluor 647 goat anti-mouse (Invitrogen, A21235) or anti-53BP1 (Novus, NB100-305) followed by Alexa Fluor 647 goat anti-rabbit (Invitrogen, A21244) were used to detect DNA damage foci, and Hoechst (Thermo Fischer Scientific, 3258) was used to stain nuclei.

The cells were imaged with a 100×/1.4 NA oil immersion objective lens (for γH2AX) or with a 63×/1.4 NA oil immersion objective lens (for 53BP1) on a spinning disk confocal microscope featuring a Zeiss AxioVert 200M inverted stand coupled to a Yokogawa CSU-22 confocal scanhead and controlled with MetaMorph 7.10. The excitation was achieved with semiconductor lasers enclosed in an Andor ILE laser launch, and the emission was recorded with a Hamamatsu Orca-ER cooled CCD camera. The fluorescence from Hoechst, GFP, and Alexa647 was excited with 405-, 488-, and 642-nm laser lines, respectively, and the emission from each channel was detected with 450/50-, 525/50-, and 700/75-nm filters, respectively. The digital resolution of the images was 0.0572 μm/pixel, and z-stacks were acquired with an axial step size of 0.5 μm.

Z-stack TIF images of γH2AX and nuclear Hoechst stain were segmented using the Cellpose algorithm [51] on maximal projections (*Z*-axis) of the 405-nm Hoechst channel images. Channel images of γH2AX foci were segmented and measured using the Laplacian of Gaussian (LoG) filter. Images were maximally projected along the *Z*-axis and subjected to the gaussian_laplace filter from the Python scipy package (sigma = 3). To identify foci, an automatic threshold was then set as 1.5 SD above the mean value of the LoG image. The image was binarized by this threshold and subjected to morphological opening to remove noise with a 3 × 3 selection element filled with 1′s. Foci were identified by the label function and measured by the ‘regionprops’ function from the scikit-image Python package. Only foci that localized in the nucleus (via the nuclear segmentation described above) were measured. The size and mean intensity of the foci were tabulated for analysis.

Cells were plated as previously described for immunofluorescence of APE1 nuclear/nucleolar localization in mESC v6.5 and C16. Anti-APE1 (Novus, NB100-101) in 5% BSA was used, followed by Alexa Fluor 647 goat anti-rabbit (Invitrogen, A21244) and Hoechst (Thermo Fisher Scientific, 3258). Immunofluorescence for APE1 in hESC H1 followed the same protocol. The LSM 980 microscope with the Airyscan detector (ZEISS) was used for image acquisition. Images were then processed using Fiji/ImageJ2v2.14.0/1.54f. Briefly, two regions of interest (ROIs) of similar areas per cell, one in the nucleolus and one in the nucleoplasm, were manually selected based on the Hoechst stain, and the APE1-specific antibody signal was measured. The mean values obtained were normalized based on nucleoplasmic intensity.

### EU nascent RNA labelling, immunofluorescence, and analysis

Here, 1 × 10^5^ cells were plated in a glass bottom 24-well plate as described in the previous paragraph. The following day, cells were treated with 50 μM CDDP for 3 and 3.5 h, with 0.5 mM MMS for 6 h, or 1 μM CX5461 for 10 and 15 min. 5-Ethynyl Uridine (EU) was added to the treated cells 5 min prior to the endpoint at a concentration of 2.5 mM. Following EU labelling, cells were fixed with 4% PFA (VWR, AA47377) for 20 min, permeabilized for 5 min with 0.5% Triton X-100 (Sigma, X100) in PBS, and blocked with 5% IgG-free BSA (VWR, 102643-516) in PBS. Anti-UBF (Santa Cruz, sc-13125) and anti-FIB1 (Abcam, ab5821) were used to detect the fibrillar centre and dense fibrillar component, respectively, followed by Alexa Fluor 488 goat anti-mouse (Invitrogen, A11029) and Alexa Fluor 647 goat anti-rabbit (Invitrogen, A21244). EU was then labelled with the Click-iT RNA Alexa Fluor 594 (Thermo Fisher Scientific, C10330) following manufacturer’s instructions, and nuclei were stained with Hoechst (Thermo Fisher Scientific, 3258). Images were taken with a 63× objective of a Zeiss LSM 980 with Airyscan 2 laser scanning confocal microscope.

Dense fibrillar component (DFC) and fibrillar center (FC) segmentation was performed as follows. Nuclei were segmented and labelled in 3D using the Hoechst channel with a Cellpose [51, 52] nuclei model retrained on a subset of representative images from the dataset. Within the resulting nuclear masks, the DFC complexes were detected using an Otsu threshold on the FIB 647 channel, followed by a binary opening mask to refine the regions.

FCs localized to the DFC compartments were segmented from the 488 channel using a watershed algorithm initialized on peak maxima identified with a difference of Gaussian blob detection algorithm and using an Otsu threshold as stop criteria. The maxima within the FC were filtered from background puncta by selecting only the peaks with values deviating 1 SD above the mean value of the maxima in the nucleoplasm.

The amount of nascent RNA within the FCs was measured by calculating the mean intensity of the EU 598 channel within the segmented FCs per cell. The pipeline for nascent RNA quantification was implemented in Python 3.10 using the scikit-image library [53].

### Live-cell imaging and analysis

Here, 1 × 10^6^ cells were plated in a glass bottom six-well plate (Cellvis, P06-1.5H-N) pre-treated with 5 μg/ml of poly-l-ornithine (Sigma, P4957) at 37°C for at least 1 h followed by 5 μg/ml of laminin (Corning, 354 232) overnight at 37°C. Cells were imaged at least 5 h after being plated with an Andor Revolution Spinning Disk Confocal microscope (Oxford Instruments) equipped with a Yokogawa CSU-X1 scanhead attached to a Nikon Ti-E inverted stand. Images were recorded with an Andor Zyla 5.5 sCMOS camera and a Nikon Plan Apo 100×/1.4 NA Ph3 oil immersion objective lens, delivering a digital resolution of 0.070 μm/px. Phase contrast microscopy with a Phase 3 mask was used for nucleolar visualization and the tagged APE1 fluorescence was excited with a 488-nm laser (Andor ILE) and detected through a combination of a 405/488/568/647 dichroic mirror and a 525/50-nm emission filter. The microscope was controlled with Metamorph 7.8 (Molecular Devices, LLC) acquisition software.

An image was recorded before the treatment, and then cells were treated either with 50 μM CDDP, 0.5 mM MMS, or 1 μM CX5461 and imaged in a time series to monitor the effects. For CDDP and MMS, the time series of the treatment effects started after 10 min from the treatment time, and cells were imaged for a total of 6 h with a 5-min interval. The time-lapse for the cells treated with CX5461 started 6 min after the treatment time for a lapse of 2 h with an interval of 3 min. The cells were kept at 37°C, 95% humidity, and 5% CO_2_ with a stage-top incubation system (Pathology Devices). Perfect Focus was engaged throughout the time course of the experiment to maintain optimal focusing of the cells. Time-lapse fluorescence images were flat-field corrected prior to any further processing.

The effect of each treatment was evaluated by analysing the nucleoli-to-nucleoplasm intensity ratio of each nucleolus over time. This required segmenting the nuclei and nucleoli for each cell, which was performed with an analysis pipeline written in Python that combines several Ilastik [54] projects for pixel classification and object detection.

Nuclei segmentation masks were obtained from the fluorescence channel and applied to the phase contrast images to isolate the nuclear bodies from the cytoplasm and other structures present in the images. Then a pixel classifier was trained to detect the nucleoli from the masked phase contrast images, and an object classifier was trained to exclude dividing and dying cells from the analysis. The segmentation masks of the nucleoli were used to calculate their mean fluorescence intensity over time, and the resulting masks from the subtraction of the nucleoli and the nuclei masks allowed quantifying the intensity in the nucleoplasm. The quantification was performed on the fluorescence images, where each nucleolus was associated with its corresponding nucleoplasm parent body to calculate the nucleoli to nucleoplasm intensity ratio.

The statistical significance of the change in the nucleoli-to-nucleoplasm fluorescence intensity ratio between the control and the treated cells was evaluated using a two-way repeated measurements ANOVA, being time and group of the independent variables. Tukey HSD was used for postdoc analysis to identify the timepoint where the effect of the studied drugs became significant.

### APE1 protein purification

DNA encoding the genes of interest (APE1^WT^, APE1^NΔ33^, APE1^K4pleA^, and APE1^K4pleR^) was cloned into a MAT-tag backbone [24] at the C-terminus of the protein. The base vector was engineered to include a linker and mEGFP tag in between the APE1 protein and the tag. Gibson Assembly Kit was used to insert these sequences in-frame. All expression constructs were sequenced through Plasmidsaurus to ensure sequence identity and proper in-frame addition of the different components. For protein expression, plasmids were transformed into LOBSTR cells (gift of Chessman Lab) and grown as follows. A fresh bacterial colony was inoculated into 200 ml LB media containing ampicillin and grown overnight at 37°C. A total of 30 ml of the overnight culture was diluted in 500 ml pre-warmed LB with freshly added ampicillin and grown for 2 h at 37°C. Isopropyl β-D-1-thiogalactopyranoside (IPTG) was added to 1 mM, and growth continued for 3 to 4 h. Cells were collected by centrifugation at 3000 rpm in Sorval RC 6 + for 10 min and stored frozen at −80°C.

Pellets were resuspended in 15 ml of lysis buffer [50 mM Tris–HCl (pH 7.5), 500 mM NaCl] containing cOmplete EDTA-free protease inhibitor (Roche, 11873580001), and sonicated for 10 cycles of 15 s on, 60 s off, 30% power of (Branson 250 sonicator with microtip). Lysates were cleared by centrifugation at 12 000 × *g* for 30 min and added to 1 ml of Ni–NTA agarose (Invitrogen, 60-0442), pre-washed three times with lysis buffer. Tubes containing the agarose lysate slurry were rotated for 2 h at 4°C. The slurry was centrifuged at 3000 rpm for 10 min in a Thermo Scientific XTR refrigerated centrifuge. The pellet was then washed and centrifuged with 5 ml of lysis buffer addition with protease inhibitors and increasing concentration of imidazole. A total of 15 μl of each fraction was run on a 12% gel, and proteins of the correct size were dialysed into storage buffer [50 mM Tris (pH 7.5), 500 mM NaCl, 10% glycerol, 1 mM dithiothreitol (DTT)]. Any precipitate after dialysis was removed by centrifugation at 3000 rpm for 10 min. Protein concentration was measured with a BCA Protein Assay Kit (Life Technologies, 23250).

### Probes and their annealing

Tetrahydrofuran (THF), DNA, DNA complementary, rPoly-U, and rG4 (Supplementary Table S2) single-strand oligos were purchased from IDT. Oligomers for DNA sequences were obtained from [55]. rPoly-U was selected as an RNA sequence not undergoing folding, as rG4 control, whereas the sequence for the predicted rG4 was obtained using QGRS Mapper [56]. 45S sequence was used to evaluate the rG4 content of rRNA. The first rG4, with the highest score encountered, used a threshold of 20 nucleotides in length, with a minimal G-group of two, localized in the 18S subunit. Running QGRS Mapper on the 18S, we found the same sequence, just shifted by six nucleotides; therefore, we decided to test it for the *in vitro* droplet experiment (Supplementary Table S3).

Upon arrival, probes were resuspended at 100 μM in RNAse-free water. All probes, besides DNA complementary, have been labelled with Cy5 at the 5′; rPoly-U and rG4 were additionally marked with a methyl-G as the last nucleotide to maintain the oligomer more stable.

Single-stranded DNA oligos were annealed (THF–DNA complementary or DNA–DNA complementary) as follows: 100 pmol of each oligo was annealed with 150 pmol of its complementary in a final volume of 40 μl in annealing buffer [10 mM Tris–HCl (pH 7.5), 10 mM MgCl_2_, 1 mM EDTA]. Annealing was performed by heating at 95°C and cooling down overnight.

RNA probes (rPoly-U and rG4) were singularly diluted 1:1 in a buffer containing KCl (final concentration of 100 mM). Samples were placed in a water bath brought to 87°C and left to cool down overnight.

### *In vitro* droplet assay and analysis

APE1 proteins underwent de-salinization and were concentrated to 20 μM using Amicon Ultra (Fisher Scientific, UFC503024) with a buffer containing 50 mM Tris–HCl, 10% glycerol, and 1 mM DTT, following manufacturer’s instructions. The 10× AP buffer (500 mM Tris–HCl, 500 mM KCl, 100 mM MgCl_2_, 10 μg/ml BSA, 0.5% Triton X-100) was diluted to a 1× solution with RNAse-free water to which APE1 proteins were added to a final concentration according to the experimental set-up. Total RNA used for assessing its influence on APE1’s ability to form droplets *in vitro* was extracted from mESC using an RNA extraction kit (Invitrogen, 12183025) following the manufacturer’s instructions.

Images of APE1-GFP droplets were analysed using a custom script in MATLAB (vR2018b). To define droplets while accounting for inhomogeneous background intensity, adaptive thresholding was applied. Images of the APE1-GFP protein channel (488 nm) were binarized with the ‘adaptthresh’ function with a sensitivity parameter of 0.4 and a neighbourhood size of 99. Following binarization, speckles in the image foreground and background were removed using ‘bwareaopen’ with a pixel size of 5. Droplet area statistics were analysed using ‘regionprops’. The partition ratio of APE1-GFP (488-nm images) was calculated per droplet by dividing the mean pixel intensity in each droplet by the mean intensity of pixels in the background. The partition ratios of Cy5-labelled DNA and RNA probes (647-nm images) were calculated similarly, using the binary mask from the 488 channel to define the droplets. Condensed fraction was calculated by dividing the total area of droplets in the image by the area of the image.

### Evaluation of rG4 structure

Circular dichroism (CD) experiments were realized at 25°C in a Jasco J-1500 CD spectrometer. Data were acquired and processed using Spectra Manager software. Quartz cuvettes contained 500 μl of 5 μM oligonucleotide samples in 1× KPi buffer (pH∼7). Three averaged spectra were acquired in the region between 220 and 400 nm with a scan speed of 30 nm/min and a response time of 1 s.

Nuclear magnetic resonance (NMR) spectra were recorded on Bruker Advance 700 MHz spectrometer equipped with a TXI (^1^H, ^13^C, ^15^N, ^2^H), 5 mm, z-gradients probe. Experiments were performed at temperatures ranging from 37°C up to 76°C, in standard 3-mm NMR tubes. The oligonucleotide samples were prepared in 1× KPi buffer supplemented with 10% D_2_O. Spectra were processed in Topspin 4.1. Pulse sequence used was zgjrwg, and water was suppressed using a watergate W5 pulse sequence with gradients and jump and return [57].

### 2D NMR HSQC spectra for APE1–rG4 binding

Isotopically (^15^N) enriched samples of APE1 were used to identify each amide residue, based on the deposed APE1 chemical shifts from BMRB (entry code: 16516). 2D NMR spectra were recorded on Bruker AVANCE NEO 700 MHz spectrometer equipped with a TXI probe. All experiments were acquired in standard 3-mm NMR tubes containing 6% D_2_O. 2D ^1^H-^15^N SOFAST HMQC NMR experiments [58] with 960 and 480 (F2 and F1, respectively) data points were used. RNA samples at one molar equivalent were used to monitor the interaction with apo-protein APE1. Only the unambiguous identified amino acids were used for assignments using the following equation:


\begin{equation*}{\mathrm{\Delta \delta }} = \sqrt{{({\mathrm{\Delta \delta H}})}^{2} + {{(0.2 {{\mathrm{\Delta \delta N}}} })}^{2}} ,\end{equation*}

where δH and δN represent the shift in both, ^1^H and ^15^N dimensions, respectively. Peaks that undergo important volume decrease (over 2/3 loss) and the most significant shifts (above the mean) were depicted on the surface of human holo-APE1 (PDB 1bix) using UCSF ChimeraX version 1.9.

### RNA electrophoretic mobility shift assay analysis

For the RNA electrophoretic mobility shift assay (REMSA) analysis, the reactions were set up in a final volume of 20 μl using the specified concentrations of APE1 recombinant proteins and 25 nM of the oligoribonucleotides, which were pre-heated at 70°C before being added to the reaction. The reactions were carried out in a buffer containing 20 mM HEPES (pH 7.5), 50 mM KCl, 0.5 μg/ml BSA, and 0.25% glycerol. After incubation for 20 min at RT, 2 μl of Orange Loading Dye (LI-COR Biosciences, 927-10100) was then added to each sample. The samples were then electrophoresed on a 4% native gel through polyacrylamide gel electrophoresis at 60 V for the first 15 min, followed by 80 V for the remaining 60 min, at 4°C in Tris-Borate-EDTA (TBE) 0.5× buffer. Gels were scanned with Odyssey CLx scanner and analysed by ImageStudio Software (LI-COR Biosciences).

### Statistical analysis

Statistical analyses were performed by using either the Student’s *t*-test or the two-way ANOVA. *P* < .05 was considered statistically significant.

## Results

### Generation and characterization of endogenous APE1 mEGFP-tagged mESCs

**Figure 1.**
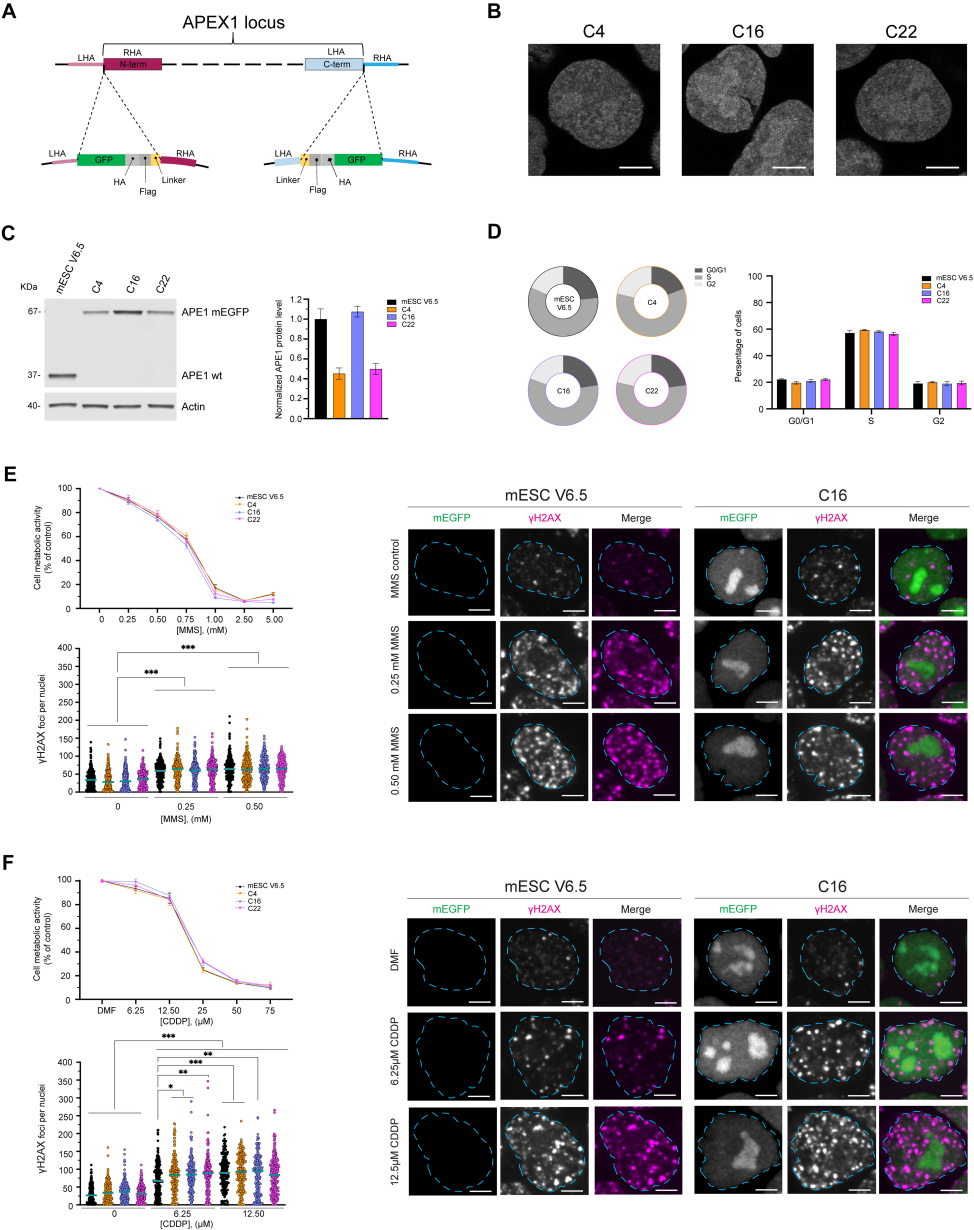
Generation and characterization of APE1 endogenous tagged mESC. Schematic representation of CRISPR–Cas9 technology used to generate the endogenously tagged mESC; N-terminus tagging of APE1 to the left and C-terminus tagging to the right (**A**). Live-cell imaging of C4, C16, and C22 clones with APE1 tagged at the C-terminus of the protein shows proper nuclear localization, with nucleolar enrichment of APE1; images were taken with a 63× objective of a Zeiss LSM 980 with Airyscan 2 laser scanning confocal microscope (scale bar 5 μm) (**B**). Representative western blot, with respective quantification analysis, performed on a biological triplicate and normalized by actin protein level, setting APE1 WT as 1; error bars represent standard error of the mean (SEM) (**C**). Cell sorting for cell cycle analysis on APE1 tagged clones, pie charts representing a single experiment are shown to the left, and a histogram with biological triplicates is shown to the right; error bars represent SEM (**D**). 3-(4,5-dimethylthiazol-2-yl)-5-(3-carboxymethoxyphenyl)-2-(4-sulfophenyl)-2H-tetrazolium (MTS) viabilities assays of cells treated with different doses of MMS for 8 h (**E**) or CDDP for 24 h (**F**), values of four biological replicates have been normalized based on control (plain media or media containing Dimethylformamide (DMF), respectively); error bars represent SEM. Staining for γH2AX on cells treated either with control (plain media), 0.25 or 0.5 mM MMS for 8 h (panel E) and control (DMF), 6.25 and 12.5 μM CDDP for 24 h (panel F) with γH2AX foci count and representative images of WT and C16 clone are shown; images are taken with 100× objective of an RPI Spinning Disk Confocal microscope, foci measurement performed on biological triplicate (two-way ANOVA statistical analysis with *P < .05, **P < .001, and ***P < .0001).

To visualize APE1 dynamic movements within living cells, avoiding non-specific effects due to overexpression strategies present in all cell models currently available for the study of this multifunctional protein, we tagged APE1 at the endogenous locus with mEGFP at either its N- or C-terminus, using the CRISPR/Cas9 system in mESCs (schematic representation of the model used is given in Fig. 1A and primers used are listed in Supplementary Table S1). To confirm successful tagging and to select cells tagged in a homozygous manner, we picked 32 colonies and confirmed successful tagging via PCR on isolated genomic DNA. From these, five homozygously tagged clones were selected for downstream analyses (Supplementary Fig. S1A). APE1 is known to have a nuclear localization with nucleolar enrichment in different cell types [12, 27, 49, 59], including hESCs (Supplementary Fig. S1B). To observe if the tagging had any effect on APE1 subcellular distribution, we performed live-cell-imaging analyses on the selected clones. We observed the expected nuclear localization for both the N- and C-terminus tagged APE1 but, interestingly, the clones tagged at the N-terminus showed a clear nucleolar depletion (Supplementary Fig. S1C). This is not a surprising effect considering that the N-terminal region of APE1 is an essential portion responsible for protein–protein and protein–RNA interactions (e.g. NPM1 and ATR) [7, 10, 22, 27, 49, 60]. For this reason, we decided to proceed only with the validation and characterization of the C-terminus tagged clones (Fig. 1B).

Another important aspect when generating endogenously tagged cell lines is that the addition of the tag(s) can alter protein expression or stability. To evaluate whether the addition of the tags might have altered the protein expression of APE1, we performed western blotting analyses, with a specific anti-APE1 antibody. We observed that one out of the three selected clones, i.e. C16, possessed comparable protein levels to the wild-type (WT) cells, whereas the other two clones, C4 and C22, showed almost half of the protein amount with respect to the WT cells (Fig. 1C). As an orthogonal approach, we evaluated the expression level of tagged APE1 by using Fluorescence-activated cell sorting (FACS) analysis exploiting the fluorescence of the mEGFP tag, which demonstrated that clones C4 and C22 had half the expression of mEGFP when compared with clone C16, consistent with the results of the western blotting analysis (Supplementary Fig. S1D). To further investigate whether the addition of the tag impaired APE1 nucleolar accumulation, we assessed its nucleolar enrichment via immunofluorescence, using the same antibody used for the western blotting. By comparing APE1 nucleolar/nucleoplasmic ratios between mESC v6.5 and C16, we observed an ∼40% increase in APE1 signal within nucleoli in both cell types (Supplementary Fig. S1E and F).

Due to its redox-regulating function on different transcription factors, APE1 is involved in cell cycle control [4]; therefore, it is of relevance to control for cell cycle dysregulation upon the addition of the tag. Consequently, we assessed if the expression of the engineered tagged-APE1, or the lower expression levels, might alter the cell cycle distribution of mESCs. By performing cell cycle analysis on sorted cells, we concluded that neither the addition of the tag nor the decrease in APE1 amounts had a major effect on the G0/G1, S, and G2 distribution of the cell cycle of mESC (Fig. 1D and Supplementary Fig. S1G).

APE1 is an essential DNA damage repair protein involved not only within the BER pathway [2, 3] but also in CDDP drug resistance [17, 61, 62]. To confirm that the addition of the tag did not alter its DNA repair activity, we evaluated cell viability as well as number of γH2AX (Fig. 1E and F) and 53BP1 foci (Supplementary Fig. S1H and I) by comparing the newly generated clones with the WT cells, exposing them to different doses of MMS or CDDP. We chose those genotoxic agents because MMS is a well-known alkylating agent, which induces DNA lesions specifically repaired by the BER pathway when used within 8 h of treatment [63, 64] and APE1 is associated with CDDP drug resistance of cells treated for 24 h [17, 61, 62, 65]. In this regard, we first evaluated cell viability via an MTS assay of WT cells and the selected clones, which showed no significant alteration in cell toxicity (Fig. 1E and 1F). To further evaluate the efficiency of the DDR of the clones, we measured the number of γH2AX and 53BP1 foci generated upon low doses of MMS (0.25 and 0.5 mM) or CDDP (6.25 and 12.5 μM) and noticed similar treatment responses comparable to the WT cells (Fig. 1E and F and Supplementary Fig. S1H and I).

Altogether, these results show that the addition of the tags at the N-terminus altered APE1 subcellular localization, whereas the C-terminal tagging did not alter either APE1 subcellular localization or DDR activity. More importantly, we developed the first non-tumoural cell model based on the expression of the endogenous APE1 protein fused with mEGFP, which allows monitoring of subcellular trafficking under physiological, non-overexpressing conditions.

### APE1 nucleolar accumulation depends on active rRNA transcription as determined by live-cell-imaging analyses

**Figure 2.**
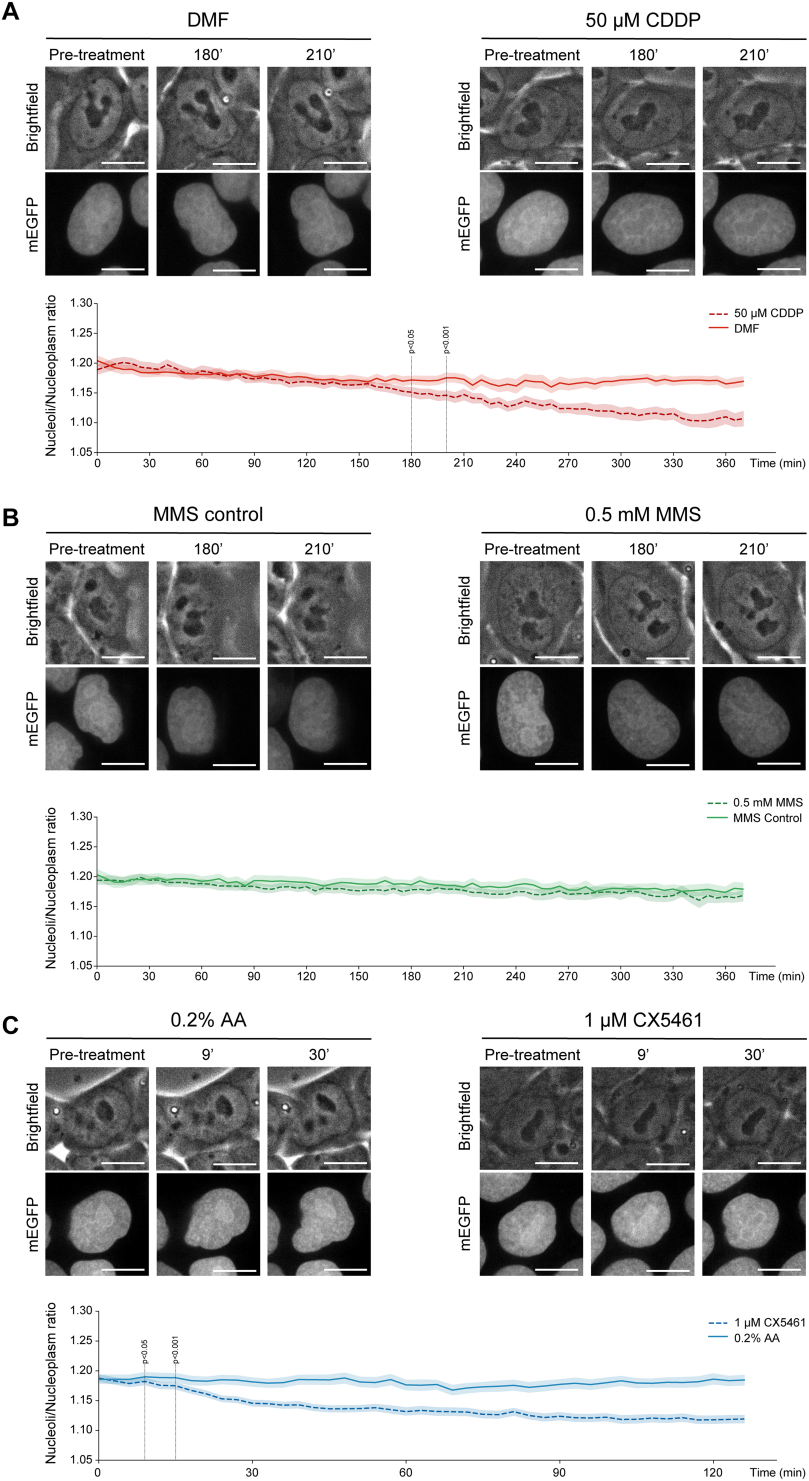
APE1 nucleolar localization is dependent on rRNA transcription. Live-cell imaging of endogenously tagged APE1 with mEGFP taken with a 100× objective of Andor Revolution Spinning Disk Confocal, FRAPPA, and TIRF system microscope. Representative images of cells before (pre-treatment), at 180 and 210 min of treatment with either 50 μM of CDDP (**A**) or 0.5 mM MMS (**B**), with respective controls, are shown; nucleoli/nucleoplasm ratio of tagged APE1 signal over time is shown on the graph below the representative images; measurements acquired for at least three different fields of the same well with the experiment repeated four times, over 150 nuclei were considered for the analysis of each treatment. Representative images of cells before (pre-treatment), at 9 and 30 min of treatment with 1 μM of CX5461 (**C**) and its control (0.2% acetic acid) are shown; nucleoli/nucleoplasm ratio of tagged APE1 signal over time is shown on the graph below representative images; measurements acquired for at least four different fields of the same well with the experiment repeated four times, over 150 nuclei were considered for the analysis (scale bar of 5 μm).

APE1 subcellular localization, though poorly understood, is very important for its activities and is altered in different cancer cells [14–16]. Our lab previously demonstrated that CDDP treatment affects APE1 nucleolar localization using high concentration (100 μM) for a short time (6 h) under temporally fixed conditions [25]. Here, we tested whether we were able to recapitulate APE1 nucleolar depletion with our newly generated cell model and track, continuously through live-cell imaging, the nucleolar emptying of the protein, which, to the best of our knowledge, has never been done before. Previous publications showed that 50 μM of CDDP was sufficient to disrupt condensates [66, 67] and impair nucleolar functions [68, 69]. By treating cells generated and characterized in this work with 50 μM of CDDP, we observed APE1 nucleolar depletion starting at 180 min, becoming significantly visible at 210 min (Fig. 2A and Supplementary Video S1A and B). To further exploit our model and test the possibility of whether other genotoxic stresses are able to disrupt APE1 nucleolar enrichment, we treated cells with 0.5 mM of MMS, which generates base lesions specifically repaired through BER [70] and observed no alteration of APE1 subcellular localization within the 6 h of acquisition (Fig. 2B and Supplementary Video S1C and D). Treating cells with 50 μM CDDP for up to 6 h did not affect cell viability (Supplementary Fig. S2A) but due to the MMS toxicity and the fact that cells started to die (Supplementary Fig. S2B), we could not extend the assay for longer timepoints. Aware of the impact that CDDP has on nucleolar functions, especially that it inhibits rRNA synthesis [68, 69, 71–75], and with regard to APE1 relevance in rRNA processing [7], we hypothesised that active rRNA transcription is essential for APE1 nucleolar accumulation. Therefore, we tracked APE1 subcellular localization of cells treated with CX5461, a clinically used RNA polymerase I (Pol I) inhibitor. As expected, we observed the same APE1 nuclear re-distribution similar to CDDP, with the first statistically significant difference observable after 9 min from the start of the treatment and being clearly visible after 30 min (Fig. 2C and Supplementary Video S1E and F).

**Figure 3.**
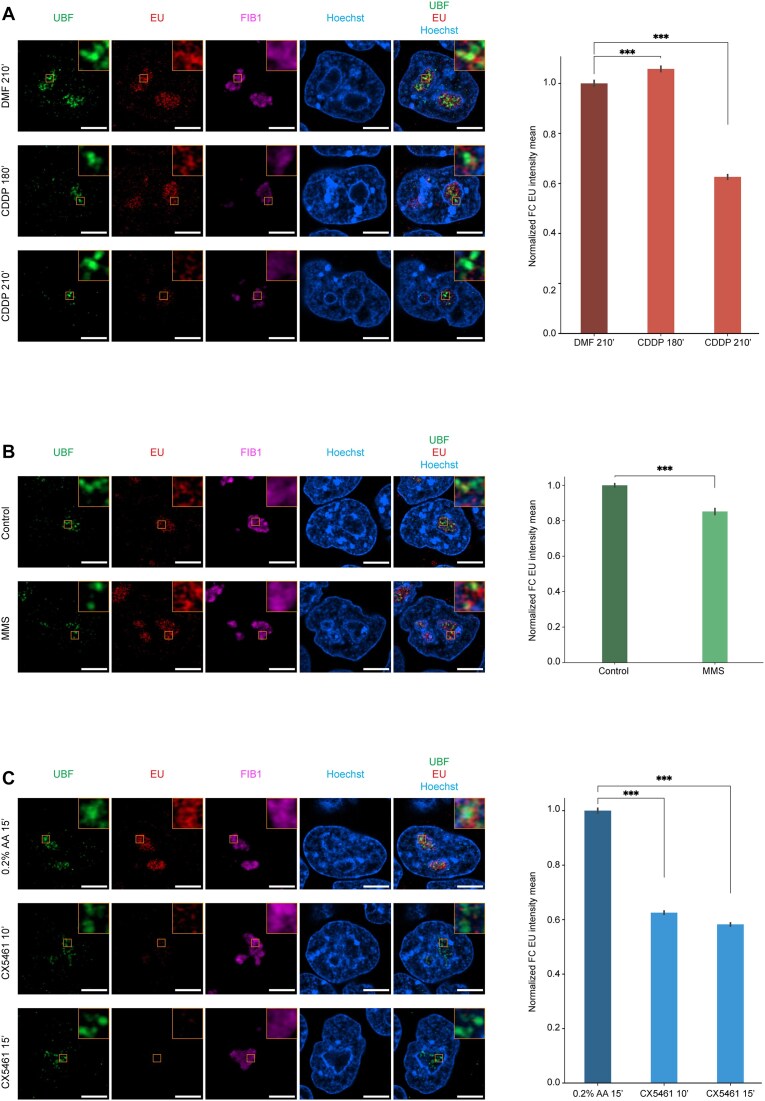
EU labelling shows decreased nucleolar RNA signal upon CDDP and CX5461 treatments. Immunofluorescence images of nascent RNA within the fibrillar centre of nucleoli upon 50 μM CDDP for 180 and 210 min (**A**), 0.5 mM MMS for 360 min (**B**), and 1 μM CX5461 for 10 and 15 min (**C**) to the left and normalized EU measurement within the fibrillar component to the right. UBF was selected to mark the nucleolar region responsible for rRNA synthesis, nascent RNA (labelled as EU), FIB1 was used to mark the dense fibrillar component surrounding the fibrillar centre, and Hoechst to delimit nuclei. Images were taken with a 63× objective of a Zeiss LSM 980 with Airyscan 2 laser scanning confocal microscope (scale bar 5 μm). Over 145 nuclei were analysed per condition (175 for DMF, 208 for 180′ CDDP, 149 for 210′ CDDP, 177 for MMS control, 186 for MMS treated, 187 for 0.2% acetic acid - AA, 222 for 10′ CX5461, and 189 for 15′ CX5461). Student’s *t*-test was used for statistical analysis with ***P < .0001.

By labelling nascent RNA with EU, we assessed the inhibition of rRNA synthesis in our experimental condition (Fig. 3). As reported in the literature, CDDP is known to cause Pol I inhibition [68, 69, 75]. This was recapitulated in mESC treated with 50 μM of CDDP within 180 and 210 min (Fig. 3A) where we observed a 40% decrease in nascent RNA at the latest timepoint. MMS treatment showed the opposite, only a minimal reduction of nascent RNA (Fig. 3B). A much faster transcriptional inhibition occurs when using CX5461, where we observed a 40% reduction in nascent RNA at 10′ from the treatment (Fig. 3C). A similar timepoint was observed by Mars *et al.* where they described rRNA synthesis inhibition within 15 min from the start of the treatment with 1 μM CX5461 [76]. Altogether these results show that not all genotoxic stress-induced DNA damages are able to promote APE1 exit from the nucleolus. Noticeably, treatment with CDDP or inhibition of RNA Pol I-mediated rRNA transcription can cause APE1 re-localization outside of the nucleolus and within the nucleoplasm, suggesting an important role of active rRNA transcription in APE1 nucleolar accumulation.

### rG4 present in rRNA promotes APE1 droplet-forming ability, which is mediated by critical lysine residues

**Figure 4.**
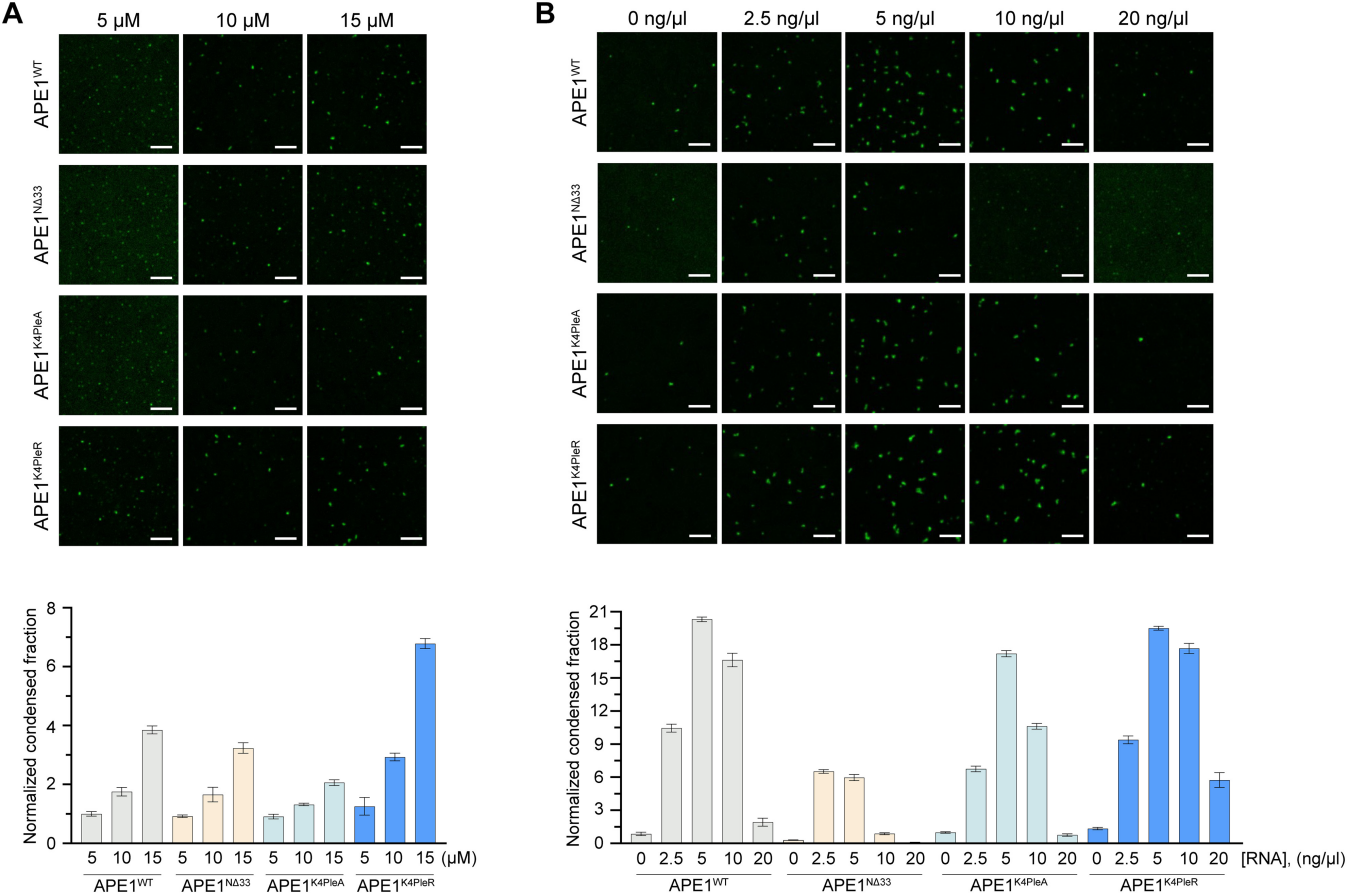
RNA influence on *in vitro*-purified APE1-mEGFP protein droplet formation. Representative images with background subtraction (top panel) of *in vitro* droplet assay, with respective normalized quantification (bottom panel) of APE1 droplets. The 5, 10, and 15 μM of APE1 proteins (APE1^WT^, APE1^NΔ33^, APE1^K4pleA^, and APE1^K4pleR^) with respective normalized measurements of condensed fraction (**A**). The 10 μM of APE1 proteins (APE1^WT^, APE1^NΔ33^, APE1^K4pleA^, and APE1^K4pleR^) in combination with 0, 2.5, 5, 10, and 20 ng/μl of total RNA with respective normalized measurement of condensed fraction (**B**). Every image shows a 5-μm scale bar. The experiment was performed twice with similar trends; images and analysis shown are of a single experiment.

Considering the recently published evidence that APE1 forms condensates in the nucleolus, promoting DDR and that it forms droplets in an *in vitro* droplet assay [49], we investigated whether we were able to recapitulate those findings using a buffer normally used for APE1 enzymatic activity *in vitro* (AP buffer) [7, 77]. Furthermore, we assessed if different APE1 mutants (APE1^NΔ33^, APE1^K4pleA^, and APE1^K4pleR^) retain the ability to form *in vitro* droplets similar to the WT protein (APE1^WT^) (Fig. 4). The N-terminal region of APE1 is known to play essential biological roles, such as in protein–protein [7, 22, 27, 49, 60] and protein–RNA interaction [7, 10, 22]. In particular, PTMs occurring in this region are known to alter APE1 physiological functions: the deletion of the first 33 amino acids can be considered a naturally occurring PTM within the cytoplasm [78] and acetylation of lysines in positions 27, 31, 32, and 35 is critical for the cellular response to genotoxic treatments [22, 23]. For this reason, we expressed and purified the protein lacking the first 33 amino acids (APE1^NΔ33^) and the previously characterized mutants: APE1^K4pleA^, in which the four lysines have been substituted with alanine or APE1^K4pleR^, in which the four lysines have been substituted with arginine. We selected these mutants due to our previous work showing that the charges provided by the lysines are relevant for APE1 nucleolar localization and RNA binding [10, 22].

Using metapredict, a deep-learning consensus predictor of intrinsic disorder and protein structure [79, 80], we observed that APE1^WT^ possesses a 60-amino acid-long IDR at its N-terminus [1] and that the disorder/order prediction is not altered in the mutated sequences (Supplementary Fig. S3). By performing an *in vitro* droplet assay with different purified protein concentrations, we observed that all the proteins were able to form droplets within a range from 5 to 15 μM, where APE1^K4pleR^ was the protein with the highest tendency to form droplets, followed by APE1^WT^. As expected, APE1^K4pleA^ and APE1^NΔ33^ demonstrated a reduced ability to form droplets *in vitro* when compared with APE1^WT^, especially at higher concentrations (Fig. 4A). In addition to protein concentration, another parameter that can influence the ability of a protein to form droplets *in vitro* is the presence of RNA or DNA molecules in a concentration-dependent manner [81–84]. Since APE1 is a protein with a positive net charge and RNA is a negatively charged biomolecule, low levels of RNA can enhance APE1 condensate formation due to favourable interactions between oppositely charged species. An increase in the overall negative charges due to higher RNA levels, beyond the point where the opposite charges compensate each other, can lead to a repulsion between the negatively charged RNA molecules, resulting in a decreased condensates formation, consistent with much published evidence [44, 46, 47, 85–88]. We extracted total RNA, which is mostly constituted by rRNA, from mESCs and performed *in vitro* droplet assays with increasing concentrations of RNA. As expected, we observed that the ability of APE1 to form droplets was affected in a dose-dependent manner by the presence of RNA, with a maximum increase at 5 ng/μl of RNA. In addition to this observation, we also noted that APE1^WT^ and APE1^K4pleR^, known to directly bind RNA, promoted better droplet formation when compared with APE1^K4pleA^ and APE1^NΔ33^, which lose their ability to directly bind RNA [22]. Surprisingly, APE1^K4pleA^ and APE1^NΔ33^ were also influenced by the addition of RNA, showing an RNA-dependent change in their condensed fraction (Fig. 4B).

**Figure 5.**
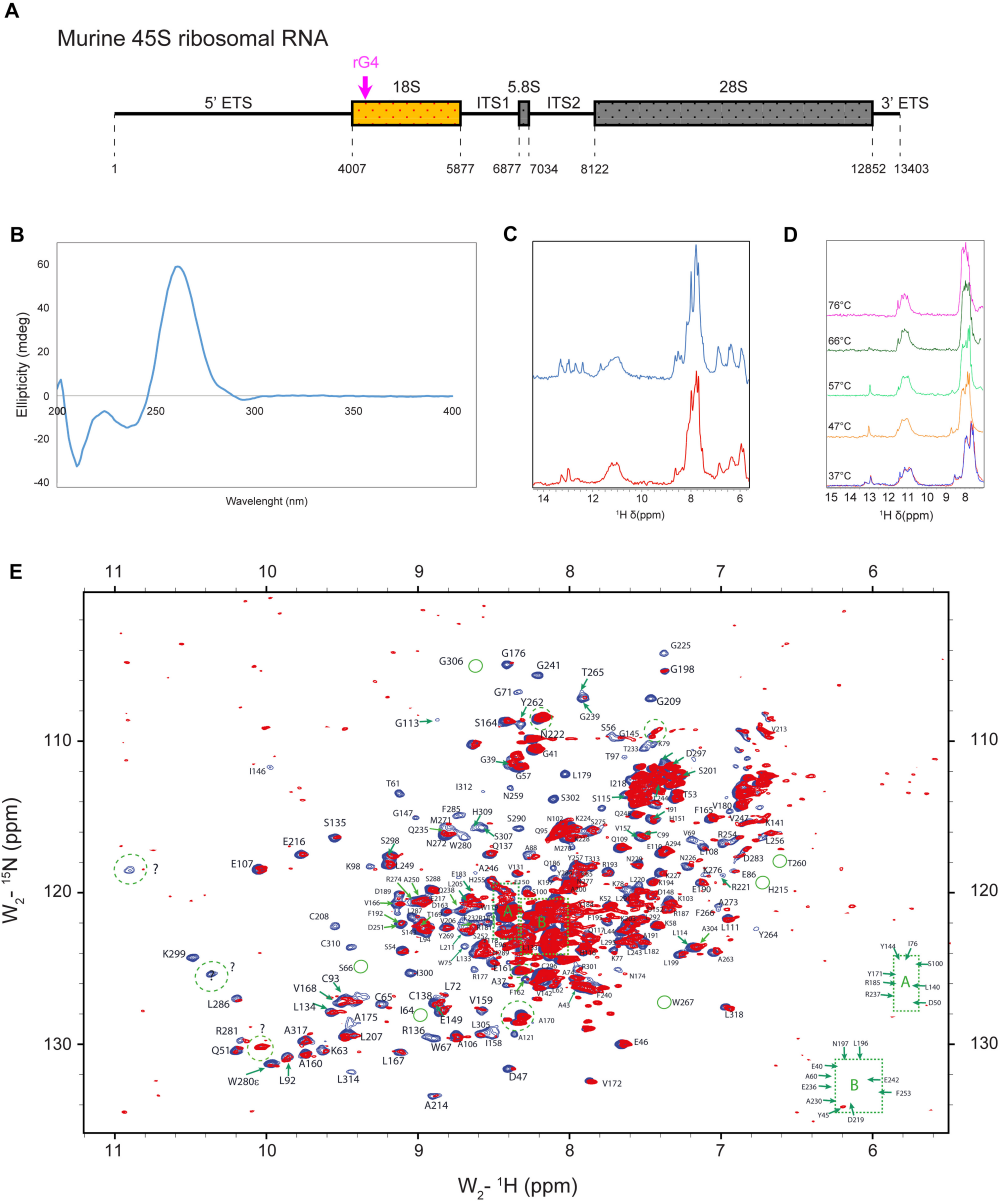
Structural analysis of the rG4 probe and APE1–rG4 protein–RNA complex. Schematic representation of the murine rRNA (45S), its components, and the location (magenta arrow) of the predicted rG4 used for this study (**A**). CD spectrum of 10 μM rG4 in KPi buffer at 25°C, evidencing a parallel ensemble of structures (**B**). The 1H spectra of 90 μM rG4 in KPi buffer at 37°C and 47°C, blue and red, respectively. Higher temperatures show the disappearance of some peaks in the imino region, indicating some unspecific interaction between RNA conformers. A polymorphic ensemble of conformers can be seen in the rG4 imino signal, between 10 and 12 (**C**). Temperature ramp spectra of 1D ^1^H NMR for rG4 oligonucleotide (**D**). Spectra were recorded at a temperature from 37°C (red spectra), to 76°C (magenta), and then a return back to 37°C (blue) overlay on the top of the starting point (red). Overlay of 2D ^15^N-^1^H NMR heteronuclear single quantum correlation (HSQC) spectra (blue) from APE1 backbone amide resonances (∼50 μM) in KPi buffer (25°C) is reported (**E**).

APE1 is emerging as an important RNA processing protein [2, 5, 8, 89], particularly implicated in rRNA processing [7, 22]. Considering our hypothesis that active transcription of rRNA is the key element necessary to keep APE1 enriched within the nucleolus and that rRNA is predicted to form several rG4s (Supplementary Table S3), we tested the influence of a putative rG4 on droplet formation for all the APE1 proteins mentioned above. First, we assessed how many rG4s were predicted to form in the murine 45S rRNA. Using QGRS Mapper, we found 120 sequences: 31 in the 5′ ETS, 8 in the 18S, 10 in the ITS1, 0 in the 5.8S, 13 in the ITS2, 49 in the 28S, and 9 in the 3′ ETS (Supplementary Table S3). To investigate the role of rG4 on APE1 droplet-forming ability, we chose a 20-mer sequence with the highest score derived from the 18S (sequences are shown in Supplementary Table S3 and location in the 45S rRNA is depicted in Fig. 5A) and compared it with a 20-nucleotide-long rPoly-U sequence (sequences are shown in Supplementary Table S2). CD and NMR assays were conducted to confirm that the 20-mer selected forms G4 (Fig. 5B and C). To check whether the rG4 sequence was prone to form multiple conformers visible in the imino region, we performed an additional 1D ^1^H NMR melting profile (Fig. 5D) with a new sample from a different provider. In addition, the samples were annealed five times to decrease, as much as possible, the number of different conformers. We performed a temperature jump from 37°C (red spectra), up to 76°C (magenta), and then a return back to 37°C (blue) overlay on top of the starting point (red). As we can observe in red spectra, some small visible peaks almost disappear at 57°C. At this temperature, the ratio of the peak at ∼13 ppm to the peaks at ∼11 ppm (G4 iminos) remains constant, indicating an internal (loop) G-C base-pairing formation. Only when the temperature reaches values closer to the *T*_M_, (between 66°C and 76°C), we do observe a complete dissolution of canonical Watson–Crick base pairing.

Electrophoretic mobility shift assay (EMSA) analysis was then used to confirm APE1’s ability to bind this sequence, showing the formation of distinct high-affinity and metastable protein–RNA complexes only with the rG4 but not with the rPoly-U, as expected (Supplementary Fig. S4A). Interestingly, the APE1^NΔ33^ completely lost the ability to bind to the rG4 in comparison to the APE1^WT^ and APE1^K4pleA^ proteins, confirming the importance of the N-terminal IDR for stable binding (Supplementary Fig. S4B).

Then, 2D NMR measurements were performed to further prove the specificity of APE1–rG4 binding, which revealed that some residues suffered a significant peak-volume reduction without important chemical shift variations (Fig. 5E). This type of peak-induced alteration is characteristic of intermediate exchange events occurring at those specific residues that may be the primary binding site. This effect likely arises from multiple binding modes and increased correlation time, leading to peak broadening or total disappearance. Residues affected include several basic residues (K63, K98, R136, R177, R221, R254, K276, and K299), aromatic residues (W67, W75, F266, W280, F285, and H309), and many others. Some of those peaks that are unambiguously identified and disappeared are well conserved and located in the binding site for the nuclease activity, such as C65, H309, C208, N274, and C310. Overall, important conserved residues that are known to bind RNA have their respective spectra densities severely diminished, indicating a medium-to-strong binding scale (hundreds of nM to just tens of nM). See Supplementary Fig. S5 for APE1 structure model representation.

Since APE1 is mostly known as a DDR enzyme [3, 11, 90], we also wanted to assess whether DNA can partition into APE1 droplets. To do so, we used two DNA sequences, one containing the AP site-mimicking THF and one unmodified control (respectively listed as THF and DNA in Supplementary Table S2). To be able to track the probe localization and assess whether they can partition into APE1 droplets, we designed them with a Cy5 label.

**Figure 6.**
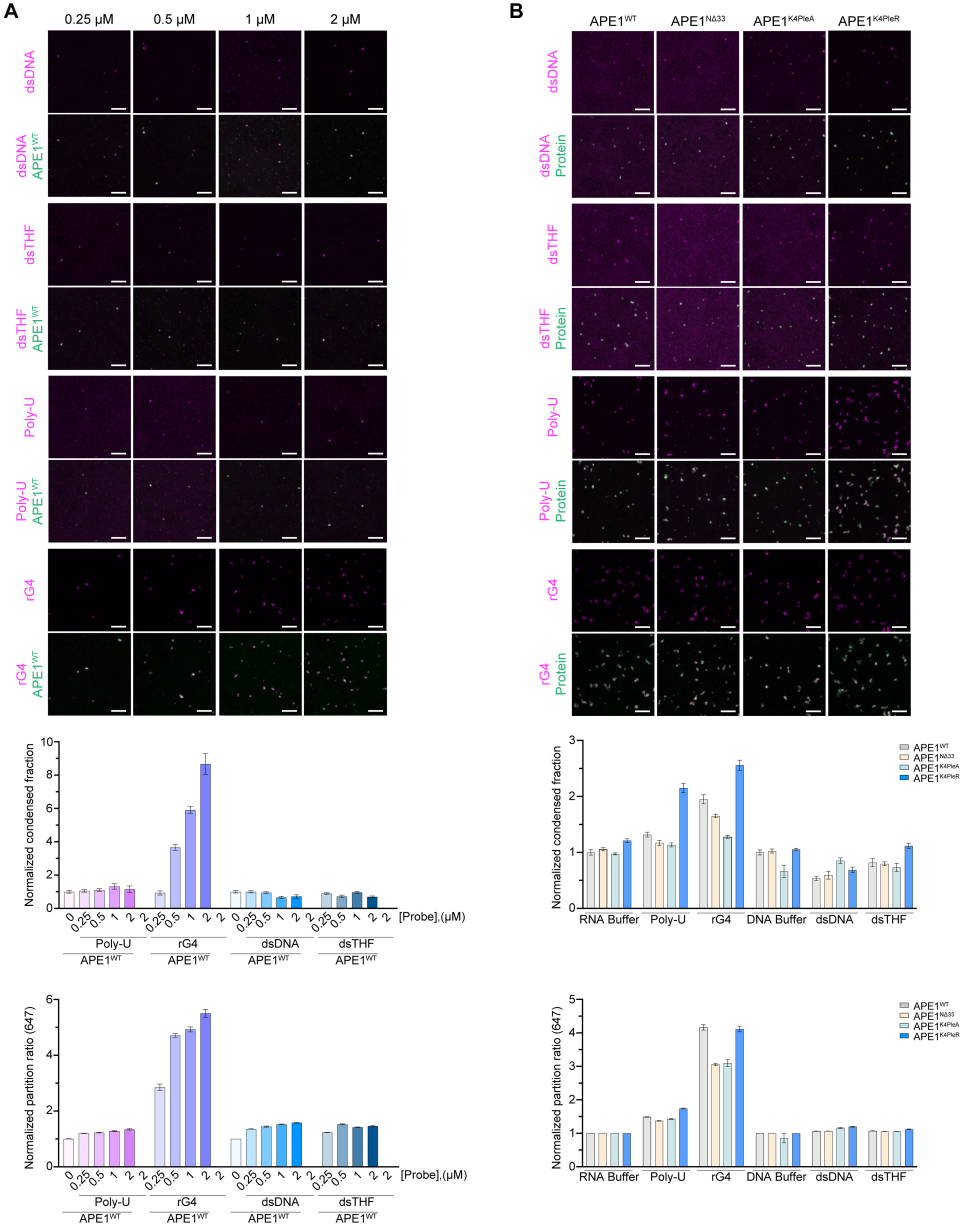
rG4 promotes *in vitro*-purified APE1-mEGFP protein droplet formation. Representative images with background subtraction (top panel) of *in vitro* droplet assay, respective normalized quantification of APE1 droplets, and nucleic acid partition into APE1 droplets are shown in the bottom panels. The 10 μM of APE1^WT^ with different concentrations (0, 0.25, 0.5, 1, and 2 μM) of probes (dsDNA, dsTHF, rPoly-U, and rG4), respective normalized measurements of condensed fraction, and partition ratio are shown (**A**). The 10 μM of the different proteins (APE1^WT^, APE1^NΔ33^, APE1^K4pleA^, and APE1^K4pleR^) with 0.5 μM of the different probes (dsDNA, dsTHF, rPoly-U, and rG4), respective measurements of condensed fraction, and partition ratio are plotted on the histograms (**B**). Scale bar of 5 μm. The experiment was performed in duplicate and showed the same trend; the images and analysis shown are of a single experiment.

To test whether those probes [double-stranded DNA (dsDNA), double-stranded THF (dsTHF), rG4, and rPoly-U] might alter APE1’s ability to form droplets *in vitro*, we performed *in vitro* droplet assay, with increasing probe concentrations using APE1^WT^. Interestingly, we observed that dsDNA and dsTHF had only a mild influence on APE1’s ability to form *in vitro* droplets using concentrations ranging from 0.25 to 2 μM. The rPoly-U probe showed a similar influence on APE1^WT^, whereas the same concentration of rG4 RNA probes improved APE1’s ability to form *in vitro* droplets (Fig. 6A). By monitoring the probe localization using the Cy5 label, we observed an enriched signal for each probe inside of APE1^WT^ droplets, suggesting their partitioning into APE1 droplets. In support of our hypothesis of rG4 promoting APE1 droplet formation, at similar probe concentrations, rG4 had a much higher ability to partition into APE1 droplets compared with all the other probes (Fig. 6). By using 0.5 μM probe concentration, we compared differences in droplet-forming ability and partitioning of APE1^WT^ with the mutants mentioned above. With this assay, we observed that (i) the presence of general RNA (rPoly-U in our case) particularly affects APE1^K4pleR^ droplet ability, (ii) the dsDNA and dsTHF probes can partition similarly between the different mutants, and (iii) rG4 can partition in droplets formed by all the tested proteins, with a higher partition ratio in APE1^WT^ and APE1^K4pleR^ (Fig. 6B). Altogether, these findings confirmed the ability of APE1 to form *in vitro* droplets in the AP buffer at concentration similar to the physiological-like one and that rG4s may improve APE1 droplet formation.

## Discussion

This work was undertaken to address some important still-pending issues concerning the study of APE1 protein, such as (i) the lack of a physiological cell model to study its functions and dynamic subcellular distribution under different stress conditions using live-cell-imaging techniques; (ii) the structural determinants responsible for APE1 nucleolar localization and the role of its IDR; and (iii) the ability of APE1 to make condensates under physiological conditions and the contribution of RNA/DNA in possibly modulating these processes. Therefore, to fulfil these aims, we here described the generation and functional characterization of the first endogenously, fluorescently tagged mESC cell line to study APE1 subcellular trafficking under physiological and genotoxic stress conditions. By using this cell model, we were able to overcome the limitations of previous studies, which mainly used overexpression cell models and immunofluorescence analyses made upon cell fixation protocols, which could generate artefacts. Using this cell line, which expresses the physiological concentration of the engineered APE1 protein, we showed that APE1 nucleolar enrichment occurs under physiological conditions requiring active rDNA transcription. By performing *in vitro* droplet assays, we provided clear evidence that phase separation, promoted by rG4 structures, is a main player in driving APE1 condensate formation at the basis of nucleolar accumulation.

The BER-associated endonuclease APE1 is essential for several biological functions, including DNA repair [3], RNA processing, and RNA quality control [91]. APE1, the only protein within the BER pathway possessing endonuclease activity, is essential for proper cell homeostasis, and its reduction or absence can drastically impair organismal viability and development [92–94]. Fine-tuning mechanisms are necessary to modulate the different functions of APE1, and it is emerging that their alterations are involved in the onset of pathological states. In particular, there is a growing amount of evidence that associates APE1 overexpression with the development of several types of cancers, including lung [17], colon [95], liver [96], prostate [97], ovarian [98], and bladder cancer [99]. In addition to that evidence, a recent study suggested that elevated APE1 protein levels are associated with increased genome instability, leading to cellular oncogenic transformation [19]. The main cellular model that approaches physiological fidelity, although imperfectly, is an inducible cancer cell model previously developed by our lab [7]. This model allowed us to regulate the suppression of endogenous APE1 while concurrently expressing the recombinant protein to a comparable degree but leaving a residual expression of the endogenous protein to levels <10% of the control [7]. Along with the need for proper APE1 expression models to help us understand its physiological cellular functions, when considering the activity of proteins in the context of phase separation mechanisms, it is to be considered that protein concentration is a critical parameter [100–102]. Due to these considerations, it will be ideal to develop effective tools to study APE1 cellular activity under conditions close to cellular physiology. For these reasons, we decided to endogenously tag the APE1 gene in mESCs, which are a non-cancerous cell model. The use of this cell model offers the additional advantage of its potential in studying APE1 functions in live-cell imaging under different conditions, throughout cellular differentiation, and, potentially, also at the organismal level due to the ability of mESCs to generate fully developed mice [103].

We observed that APE1, tagged at its C-terminus, retained its well-established nuclear localization with nucleolar enrichment [12, 13, 49, 59]; on the contrary, the N-terminally tagged protein demonstrated an altered subcellular distribution. This observation can be potentially explained by the essential role that the APE1 N-terminal domain has in regulating binding activities, as extensively demonstrated by previous work from our group [7, 10, 16, 22–26] and as recently reviewed by López *et al.* [27]. Considering that APE1 altered cellular localization can be a clue to altered protein function, we proceeded with the characterization of only C-terminus tagged clones. To assess whether the addition of the tag could alter known APE1 activities, we performed cell cycle analysis and tested cellular responses to genotoxic stimuli directly associated with APE1 DNA repair activity. All the results obtained (Fig. 1) led us to conclude that neither the tags nor the reduced protein expression level observed in the selected clone could significantly alter cellular homeostasis and DDR.

Then, we wanted to test the feasibility of tracking, in real-time, the dynamic subnuclear movements of APE1 under different genotoxic stress conditions. To do so, we treated living cells with CDDP and MMS (Fig. 2A and B). As expected, we observed APE1 nucleolar depletion upon CDDP treatment, and, interestingly, no alteration in APE1 nucleolar localization was observed upon MMS treatment. Considering the established role of different platinum-based chemotherapeutic agents, including CDDP, in inhibiting RNA Pol I transcriptional activity by generating DNA crosslinks involving G4 rDNA structures [68, 69, 71–74], we wanted to assess if rRNA neo-synthesis was required for APE1 nucleolar localization. To test this hypothesis, we treated the cells with CX5461 and observed APE1 re-localization (Fig. 2C). By monitoring nascent RNA with EU labelling, we observed a decrease in nucleolar RNA synthesis when cells were treated with CDDP and CX5461 but not with MMS (Fig. 3). With this method we noticed a significant (∼40%) decrease of nascent RNA at 210 min of CDDP treatment, which is consistent with what is described in the literature [68, 69, 75]. A similar result, with a ∼40% decrease in nascent RNA, was obtained within the first 10 min of CX5461 treatment, as previously described in the literature [76]. Despite the evidence that CDDP and CX5461 can stabilize G4 structures, they have been reported to stabilize mostly G4s in DNA [104–107] and not RNA G4. Even if both drugs could bind and stabilize RNA G4, condensates are very dynamic bodies that can change behaviour based on the environment [41, 108] and different chemotherapeutic agents can play different roles in altering condensate behaviour. From the literature, we know that CDDP influences the formation of transcriptional condensates [66] and stress granules [67], suggesting that altering the chemical composition of condensates plays an important role in proper biological activities [109]. From these results, we deduce that also under normal physiological conditions, APE1 nucleolar localization is strongly dependent on active rRNA transcription, which is in agreement with our previous observations in APE1 overexpressing cancer cell models [7, 25].

Recently, Li *et al.* showed that APE1 was able to undergo phase separation promoting ATR-Chk1 nucleolar condensate formation, emphasizing that APE1 IDR was essential for condensate formation [49]. Unfortunately, this investigation has some limitations due to the use of high, non-physiological concentrations of the protein both *in vitro* (550 μM) and in cellular (overexpression conditions) assays. In addition, the role of DNA and RNA in the *in vitro* droplet system was assessed using protein extracts, which could already be devoid of nucleic acids. To overcome these issues, we generated the mESC cell model described above, showing that APE1 is accumulated in rRNA transcription-dependent nucleolar condensates under physiological conditions. Considering that in human cells, we can find from 0.35 to 7 × 10^6^ molecules of APE1 [9] and that mammalian cells have a volume spanning from 1198 up to 2425 μm^3^ [110], we estimated that APE1 protein concentration in mammalian cells can range from about 0.2 to 10 μM. Under these considerations, the results obtained by previous studies might be affected by artefacts due to the elevated protein concentration, severely hampering the protein’s ability to undergo phase separation [100, 101, 111], leading to conclusions poorly relevant from a biological point of view.

Besides observing that APE1^WT^ is able to form droplets at the low physiological micromolar range, we also observed that deletion or charge modifications to the N-terminus IDR of APE1 can change its droplet-forming ability (Fig. 4A). Since the role of RNA in influencing phase separation and droplet-forming abilities of several proteins is widely established in the scientific community [47], we wanted to assess its effect on APE1 condensate-forming ability. For this reason, we added RNA extracted from cells to our droplet solution and observed that RNA strongly affects the droplet-forming ability of not only APE1^WT^ but also, to different extents, of all the other APE1 mutants tested (Fig. 4B). Considering that (i) total RNA extracts are usually enriched (>90%) in rRNA, (ii) both rDNA genes and rRNA have been demonstrated to be highly enriched in Gs and to form G4 structures [32, 33], (iii) G4s seem involved in several biological processes, including a newly discovered association with condensates [36], and (iv) APE1 is able to bind to G4 structures not only in DNA [28, 30] but also in RNA [112], we evaluated the influence that rG4s may have on APE1’s ability to form *in vitro* droplets. The selected rG4 sequence derived from murine 18S rRNA, based on its prediction in forming rG4, was confirmed forming rG4 via CD and NMR. Furthermore, we showed APE1’s ability to bind to the selected rG4 via 2D NMR (Fig. 5D) and EMSA (Supplementary Fig. S4A). The observed line broadening on the 2D NMR spectra suggests intermediate exchange dynamics on the NMR timescale, which is characteristic of specific binding rather than non-specific electrostatic interactions (Fig. 5D). Moreover, among those that were possible to identify, the selective disappearance or shift of signals corresponding to key conserved residues—including C65, C208, N274, S307, H309, and C310—implies that these residues are directly involved in rG4 binding or are undergoing conformational rearrangements upon interaction. The clustering of affected residues further supports the notion that APE1 engages with rG4 in a structurally defined manner, likely forming a stable complex that alters the local environment of these residues. Additionally, the dispersion of the remaining peaks suggests that the overall fold of APE1 is retained, reinforcing that rG4 binding induces localized rather than global structural perturbations. The presence of residual peaks with reduced intensity might reflect a dynamic equilibrium between the free and bound states of the protein, further corroborating the moderate-to-high affinity interaction. These residues are located near or within functionally relevant domains of APE1, particularly regions known for nucleic acid recognition and enzymatic activity, suggesting a potential overlap between rG4 binding and the catalytic or DNA-binding regions.

Interestingly, our results clearly indicate that APE1’s ability to form *in vitro* droplets and, therefore, its potential to form condensates within the cellular environment, is strongly promoted by rG4 but not by unstructured rPoly-U sequences, even when compared with APE1 natural substrates containing specific lesions (DNA containing AP sites). Despite the different binding affinity observed between the rG4 and the different APE1 mutant analysed (Supplementary Fig. S4B), the mild influence on the droplet-forming ability of the proteins when incubated with rPoly-U or the influence of the rG4 could be explained by assessing the charge of the proteins. We selected those mutants to investigate the functional role of APE1 N-terminal region and assess whether the positively charged lysines could play a role in APE1 condensate formation. As observed in Fig. 6B, the mutants that lose those positively charged residues are still able to phase separate but to a different extent. Analysing the net charge of APE1 proteins, using the Prot PI calculator (https://www.protpi.ch/Calculator/ProteinTool#Results), the isoelectric point of APE1^WT^, APE1^K4pleR^, APE1^K4pleA^, and APE1^NΔ33^ is, respectively, 7.83, 7.84, 6.64, and 6.71. Since RNA can influence the condensate behaviour of proteins [46, 47], it is reasonable to assume that APE1 mutants, despite their inability to stably bind the rRNA probe, are still subject to changes in partition ratios, when in the presence of rRNA, due to their residual charges. Based on the results obtained, it is reasonable to assume that the positive charges provided by K^27^/K^31^/K^32^/K^35^ are not only essential for the direct rRNA binding of APE1 but also for its partition behaviour within condensates, particularly within condensates enriched in rG4 such as the nucleoli. These results are partially in contrast with those obtained in previous work by others [49] and could be explained by the significantly higher concentration of protein used in this work and the DNAse and RNAse treatments used to remove nucleic acids by these authors, which could not completely remove sequence- and structure-specific oligonucleotides.

To the best of our knowledge, APE1’s ability to bind G4 structures has only been reported for DNA sequences and not for rG4. Our and others’ previous literature did not report any specific APE1 binding ability towards rG4. In fact, in Poletto *et al.* [24], we characterized the requirements for the interaction of APE1 with NPM1 and undamaged nucleic acids, showing that DNA/RNA secondary structure (only stem-loop and bulge structures, as well as the length of the double-stranded regions, without considering the possible effect of G4 structures) has an impact on APE1 binding ability. Then, in Fantini *et al.* [10], we showed that lysine residues, located in the APE1 N-terminal unstructured domain, are involved in the interaction of APE1 with both RNA and NPM1, thus supporting a competitive binding mechanism. These results suggested that protein–protein interactions and/or PTMs involving APE1 N-terminal domain may play important roles *in vivo*, such as the better coordination and fine-tuning of BER protein and function on RNA metabolism. Finally, in Poletto *et al.* [25], we unveiled a novel role for NPM1 as a modulator of the whole BER pathway by (i) controlling BER protein levels, (ii) regulating total BER capacity, and (iii) modulating the nucleolar localization of several BER enzymes. Our this paper, in addition to our recently published paper on APE1 binding to miR-92b [112], is the first one showing the importance of rG4-containing sequences being actively bound by APE1. For those reasons, we believe that our this work could significantly help to clarify, in a mechanistic way, important novel aspects of APE1 biology and far extend the comprehension of the mechanistic basis for APE1 nucleolar accumulation not only in pathologic cancer cellular models but, most interestingly, under physiologic conditions in normal, non-cancer, cell lines.

In general, the rising importance of understanding the physicochemical properties of condensate formation in DDR modulation and the ability to compartmentalize potentially harmful biological reactions [113] could be important for developing new therapeutic approaches [41, 100]. For instance, phase separation has been recently described in the case of (i) the RNA-dependent recruitment of 53BP1 and MRNIP-mediated coordination of Mre11, Rad50 and Nbs1 (MRN) complex for double strand break (DSB) repair, leading to the resolution of the damage [114, 115]; (ii) the regulation of several DDR proteins (i.e. ERCC1 and EXO1) through SENP6- and SLX4-controlled SUMOylation, allowing DDR-associated proteins to avoid excessive protein turnover and degradation [116, 117]; and (iii) the APE1-driven phase separation promoting ATR-Chk1 damage response, in which APE1 nucleolar enrichment promotes recruitment of both ATR and its activators (TopBP1 and ETAA1), leading to ATR-mediated Chk1 phosphorylation [49].

In recent years, condensates have gained a lot of attention in the scientific community, and several publications support the evidence that RNA plays a role in condensate formation [46, 47, 81, 85, 100, 109, 118]. Only recently, RNA structures, with particular emphasis on rG4, have been associated with condensate formation [36, 119–121]. Due to their abundance in guanine and their ability to form secondary structures, rG4 appears to be an advantageous scaffold for RNA-derived phase separation by allowing multivalent interactions between RNAs and RNA-binding proteins [120] not to mention that G-rich RNA was observed to have gel-like properties in 1910 (described and summarized in [85]). A possible limitation of our study is that we could not directly demonstrate the causal link between rG4 structures and condensate formation. Therefore, while additional experimental rigorous proof should be reached, altogether all our findings reinforce the overall hypothesis that APE1’s ability to undergo phase separation could be promoted by non-canonical RNA secondary structures, including rG4. Furthermore, APE1’s ability to undergo phase separation could be an important link in understanding APE1 functions beyond its DNA damage repair activity.

Overall, the results of this study suggest that the ability of APE1 to form *in vitro* and *in vivo* condensates is a physiological mechanism to compartmentalize the protein within cells that strongly depends on specific RNA structures. To this end, the ability of APE1 to recognize and process RNAs containing both AP and 8-oxo-guanine sites, alone or in combination with additional proteins through phase separation mechanisms, may represent an interesting future subject of investigation. These future studies, aiming at defining the regulatory functions of rG4s present in nuclear RNAs and understanding molecular mechanisms in RNA quality control, will open new opportunities for developing novel specific anticancer strategies.

## Supplementary Material

gkaf168_Supplemental_Files

## Data Availability

The data underlying this article are available in the article and in its online supplementary material. Additional raw data underlying this article are available in Zenodo, at https://zenodo.org/uploads/14218366.
