# Supplementary material for: APE1 condensation in nucleoli of non-cancer cells depends on rRNA transcription and forming G-quadruplex RNA structures
Source: Nucleic Acids Res. 2025 Mar 18;53(5):gkaf168. doi: 10.1093/nar/gkaf168 (PMC11915510; doi:10.1093/nar/gkaf168)
Supplement: gkaf168_Supplemental_Files [file gkaf168_supplemental_files.zip › Dall'Agnese et al SI_TEXT_Revised_20250203_FINAL.docx]

**Supplementary Information**

**APE1 condensation in nucleoli of non-cancer cells depends on rRNA transcription and forming G-quadruplex RNA structures**

Giuseppe Dall’Agnese^1,2^, Nancy M. Hannett^2^, Kalon J. Overholt^2,3^, Jesse M. Platt^2,4^, Jonathan E. Henninger^2^, Asier Marcos-Vidal^5^, Zahraa Othman^6^, Gilmar Salgado^6^, Giulia Antoniali^1,*^, Gianluca Tell^1^.*

^1^Laboratory of Molecular Biology and DNA repair, Department of Medicine, University of Udine, 33100 Udine, Italy

^2^Whitehead Institute for Biomedical Research, 455 Main Street, Cambridge, MA 02142, USA

^3^Department of Biological Engineering, Massachusetts Institute of Technology, Cambridge, MA, USA

^4^Division of Gastroenterology, Department of Medicine, Massachusetts General Hospital, Boston, MA, 02114, USA

^5^W.M. Keck Imaging Facility, Whitehead Institute for Biomedical Research, Cambridge, MA, USA

^6^ ARNA lab (Nucleic Acids: Natural and Artificial Regulations)- Inserm U1212 - CNRS UMR 5320, Bordeaux Biologie Santé Université de Bordeaux, 146. Rue Léo Saignant. BORDEAUX (33), France

*Corresponding authors:

Prof. Gianluca Tell

Laboratory of Molecular Biology and DNA repair, Department of Medicine, University of Udine, 33100 Udine, Italy

**Email: [gianluca.tell@uniud.it](mailto:gianluca.tell@uniud.it)**

Prof. Giulia Antoniali

Laboratory of Molecular Biology and DNA repair, Department of Medicine, University of Udine, 33100 Udine, Italy

**Email:** [**giulia.antoniali@uniud.it**](mailto:giulia.antoniali@uniud.it)

**Author Contributions:** G.T. together with G.A. conceived and designed the study and supervised the experiments contributing to the interpretation of the results; G.D. performed the majority of the experiments, analyzed the data, and critically contributed to the interpretation of the results; N.M.H. performed the protein expression and purification for *in vitro* droplets; K.J.O., J.E.H. and A.M.V. performed the quantification analyses; J.M.P. contributed to the strategy and generation of the endogenously tagged cells; G.D., G.A. and G.T. mainly wrote the manuscript; N.M.H., K.J.O., J.M.P., J.E.H. and A.M.V provided critical comments and suggestions and contributed to the interpretation of the results. All authors critically read and approved the final version of the manuscript.

**Competing Interest Statement:** The authors declare no competing interests.

**Keywords:** Condensates, Nucleolus, Base Excision Repair, APE1, RNA G-quadruplex.

**This PDF file includes:**

Supplementary Figures 1 to 5

Supplementary Tables 1 to 3

Supplementary Videos

**Supplementary Fig.1. Further characterization of APE1 endogenous tagged murine Embryonic Stem Cells (mESC).**

Results of the agarose gel electrophoresis performed on PCR products to validate the homozygosity of the clones (A). APE1 immunofluorescence of hESC with nuclear protein localization and nucleolar enrichment (B). Live cell imaging of the 5 selected clones tagged at the C-terminus or the N-terminus; images taken with a 63x objective of a Zeiss LSM 980 with Airyscan 2 Laser Scanning Confocal microscope, scale bar 5 μm (C). Comparative measurement of EGFP from the different clones resulting from cell sorting (D). APE1 immunofluorescence of mESC v6.5 (E) and C16 (F) with normalized nucleolar/nucleoplasmic ratios. Images were taken with a 63x objective of a Zeiss LSM 980 with Airyscan 2 Laser Scanning Confocal microscope, scale bar 5 μm. Representative curves of cell cycle stages comparing mESC v6.5 with C4, C16 and C22 clones (G). 53BP1 foci count on cells treated either with control (plain media), 0.25 mM or 0.5 mM MMS for 8 hours (H) and control (DMF), 6.25 μM and 12.5 μM CDDP for 24 hours (I). 2 way ANOVA statistical analysis performed for MMS treatment showed statistical significance with p<0.05 when comparing mESC v6.5 0 mM MMS with mESC v6.5 0.50 mM MMS, C4 0 mM MMS with mESC v6.5 0.25 mM MMS, C4 0.25 mM MMS with mESC v6.5 0.50 mM MMS, C16 0 MM MMS with C4 0.50 mM MMS, C16 0.25 mM MMS with C22 0.25 mM MMS and C22 0 mM MMS with C16 0.5 mM MMS; p<0.001 for comparisons between mESC v6.5 0 mM MMS and C4 0.5 mM MMS, C4 0.25 mM MMS with C4 0.50 mM MMS, C22 0 mM MMS with C16 0.25 mM MMS and C22 0.25 mM MMS with mESC v6.5 0.5 mM MMS; and p<0.0001 when comparing C4 0 mM MMS with C16 0.25 mM MMS, mESC v6.5 0.50 mM MMS, C4 0.50 mM MMS and C22 0.50 mM MMS, comparisons between C22 0 mM MMS and mESC v6.5 0.50 mM MMS and C4 0.50 mM MMS as well as comparing C22 0.25 mM MMS with C4 0.50 mM MMS. The same 2 way ANOVA statistical analysis was performed for CDDP treatment showing statistical significance with p<0.05 when comparing mESC v6.5 DMF with C22 DMF, mESC v6.5 6,25 μM CDDP, between mESC v6.5 12.5 μM CDDP and C4 12.5 μM CDDP, C4 6.25 μM CDDP compared to C22 6.25 μM CDDP and between C16 6.25 μM CDDP and C16 12.5 μM CDDP; p<0.001 for comparisons between C16 12.5 μM CDDP and C22 12.5 μM CDDP; and p<0.0001 when comparing mESC v6.5 DMF with C16 DMF, C4 6.25 μM CDDP, C16 6.25  μM CDDP, C22 6.25  μM CDDP, as well as with all the cells treated with 12.5  μM CDDP, with mESC v6.5 6.25 μM CDDP compared to C4 and C16 treated with 12.5 μM CDDP, between mESC v6.5 and C16 when treated with 12.5 μM CDDP, and comparing C22 6.25 μM CDDP with C16 12.5 μM CDDP, p<0.0001 was also observed when comparing C4, C16 and C22 treated with the control (DMF), and all the CDDP treated conditions. Images taken with 63x objective of an RPI Spinning Disk Confocal microscope, foci measurement performed on biological triplicate.

**Supplementary Fig.2. Viability validation on cells treated with CDDP or MMS for live cell imaging.**

MTS viabilities assays of cells treated with 50 μM CDDP (A) or 0.5 mM MMS for 6 hours (B). Values of three biological replicates have been normalized based on control (DMF or plain media respectively); error bars represent SEM.

**Supplementary Fig.3. Disorder/order prediction plots of APE1 proteins.**

Ordered-disordered prediction of APE1 proteins using metapredict (78, 79). APE1^WT^ (A); APE1^NΔ33^ (B); APE1^K4pleA^ (C) and APE1^K4pleR^ (D). The predicted IDR is shown in red.

**Supplementary Fig.4. APE1 binding to rG4 via REMSA.**

REMSA analysis with different amounts of recombinant APE1^WT^ incubated with rG4 or rPoly-U probes (25 nM). APE1 stable protein complexes are clearly visible with the rG4 probe as well as some metastable complexes distributed along the lane but not with the rPoly-U sequence (A). REMSA analysis with different amounts of recombinant APE1 mutants, APE1^WT^, APE1^ND33^ and APE1^KpleA^ incubated with rG4 (25 nM). APE1 stable protein complexes are clearly visible with the APE1^WT^, and APE1^KpleA^ as well as some metastable complexes distributed along the lane but not with APE1^ND33^. The residual lower amount of the free probe in the APE1^WT^ with respect to the APE1^KpleA^ mutant is consistent with a lower ability of this mutant to generate protein-RNA complexes (B).

**Supplementary Fig.5. APE1 binding to rG4 most affected residues by 2D NMR.**

NMR chemical shift perturbations (CSPs) of APE1 amide backbone groups, most affected upon rG4 binding, were mapped onto the structure of holo-APE1 (PDB ID:1bix). The protein is depicted in blue and the grey ribbons represent the oligonucleotide deposited on the PDB and are for illustrative purposes only. Yellow residues represent the amino acids whose peaks decreased in intensity (over 2/3 volume lost) or completely vanished. Red peaks represent the most important chemical shift deviations after the inclusion of rG4 as depicted on the 2D spectra in Figure 5E. The figure, especially panel A, helps to understand that APE1 binds rG4 near the canonical binding site observed in complexes with many different oligonucleotides reported in the literature.

**Supplementary Table 1**

List of primers used to generate the endogenously tagged APE1 cell line at the C-terminus (A) and at the N-terminus (B). Sequences in red capital letters were used as primers for PCR; lowercase, bold, letters were used as overhangs for the Gibson assembly of the plasmids; italic lowercase letters highlight the linker sequence used to space from APE1 sequence to the tag sequence; underlined, lowercase letters mark the addition of the Flag tag whereas lowercase letters mark the addition of the HA tag; guide RNA (gRNA) sequences are reported in blue. A29-A30 and A32-A33 were used to generate PCR-amplified LHA and RHA for the C-terminus tagging location, similarly, A38-A39 and A40-A41 were used for the N-terminus homology arms. A09-A31 and A19-A20 primers were used to amplify mEGFP from a plasmid for C-terminus and N-terminus respectively. List of primers used to perform PCR from genomic DNA to validate the hetero-/homo-zygosity state of the cells upon electrophoretic agarose gel assay (A71 and A72 for the N-terminus tag, A73 and A74 for the C-terminus tag) (C).

**Supplementary Table 2**

Table of predicted rG4 present in the murine 45S rRNA (left) and 18S rRNA (right) with position, length, sequence, and score using GQRS-Mapper; sequences resulting from the prediction that are 20 nucleotide long are listed in bold characters; the selected sequence, predicted to form rG4 used in the *in vitro* droplet assays is highlighted in magenta.

**Supplementary Table 3**

DNA sequences used for *in vitro* droplet assay: DNA and THF, both labelled with Cy5 fluorophore at the 5’ end of the sequences, complementary sequence to generate the double-strand DNA; RNA sequences for *in vitro* droplet assay: rPoly-U and rG4, both labelled with Cy5 fluorophore at the 5’ end of the sequences with the addition of a methyl-G at the 3’ end of the sequence.

**Supplementary Videos. Time-lapse of mESC C16 treated with different genotoxic compounds.**

Time-lapse video of cells treated with DMF, vehicle, (A) or 50 μM CDDP (B); with just media, as control, (C) or with 0.5 mM MMS (D); 0.2% acetic acid as control, (E) or RNA PolI inhibitor CX5461 (F). The time stamp is on the top right and the scale bar is 10 μm on the bottom right.
