# Supplementary figures and images for: APE1 condensation in nucleoli of non-cancer cells depends on rRNA transcription and forming G-quadruplex RNA structures
Source: Nucleic Acids Res. 2025 Mar 18;53(5):gkaf168. doi: 10.1093/nar/gkaf168 (PMC11915510; doi:10.1093/nar/gkaf168)

# Supp. Figure 1

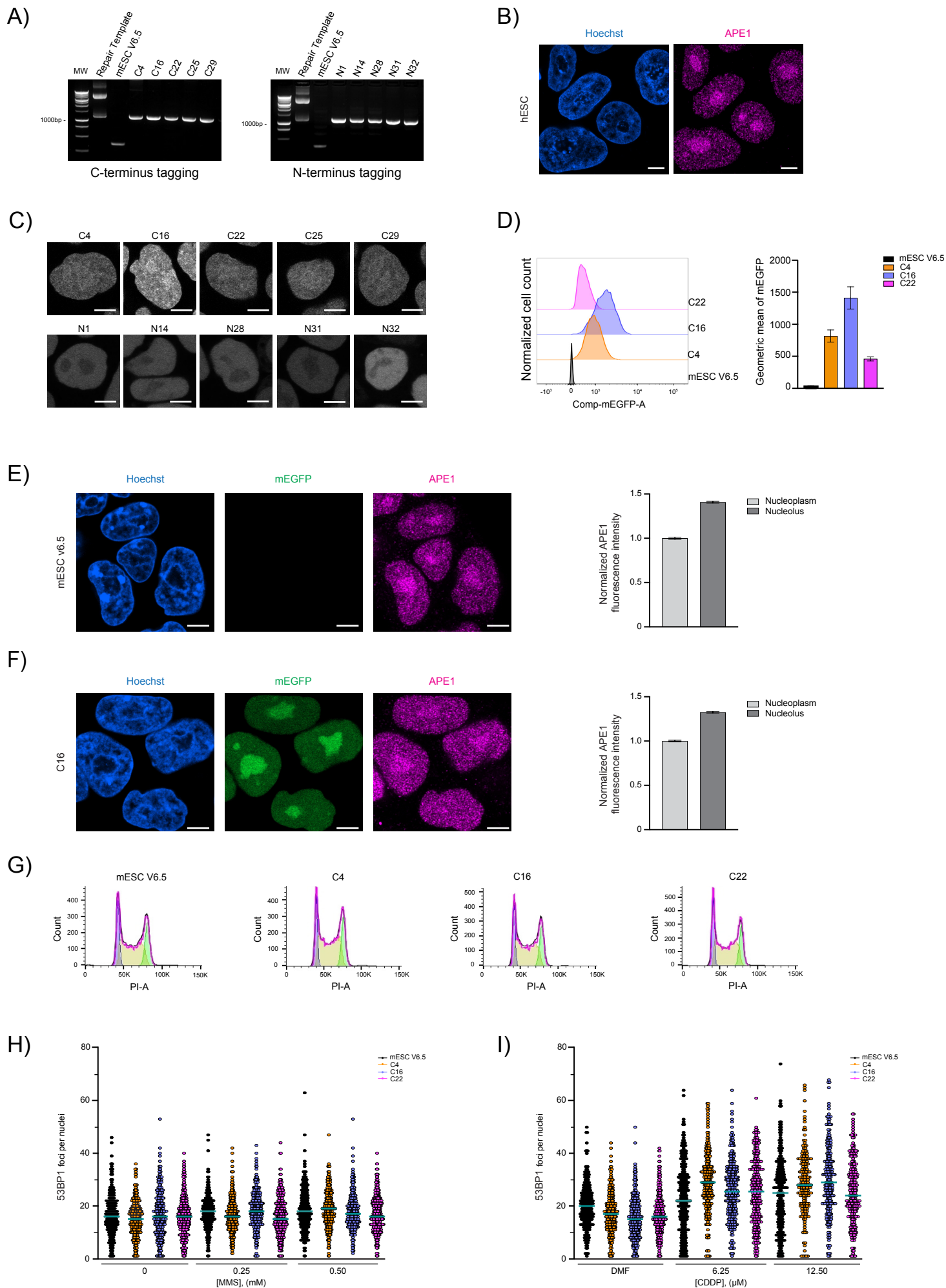

# Supp. Figure 2

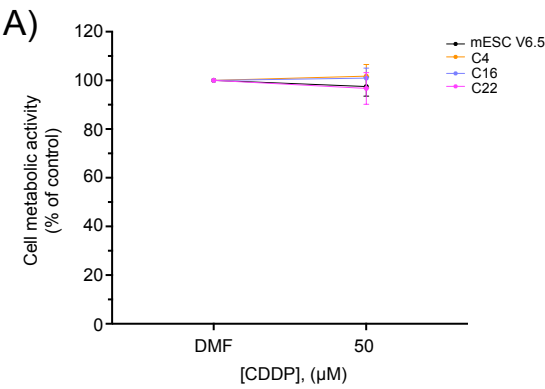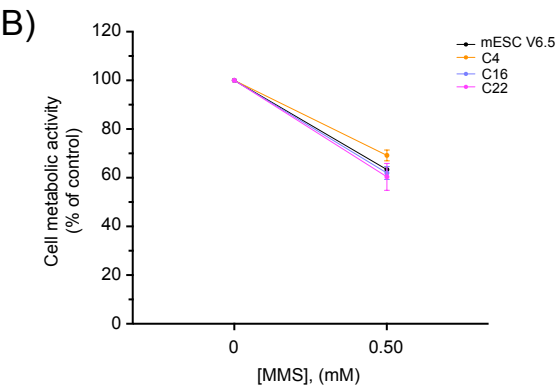

# Supp. Figure 3

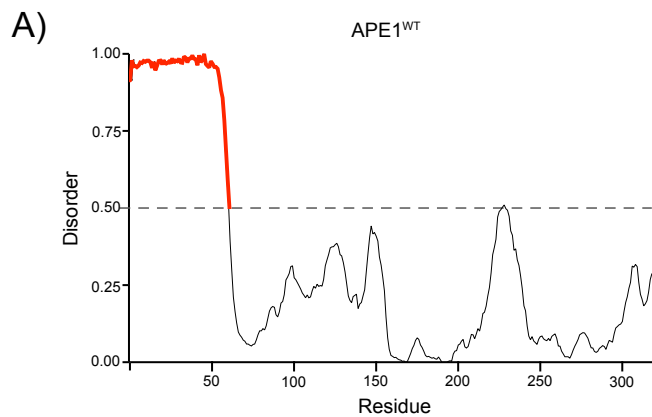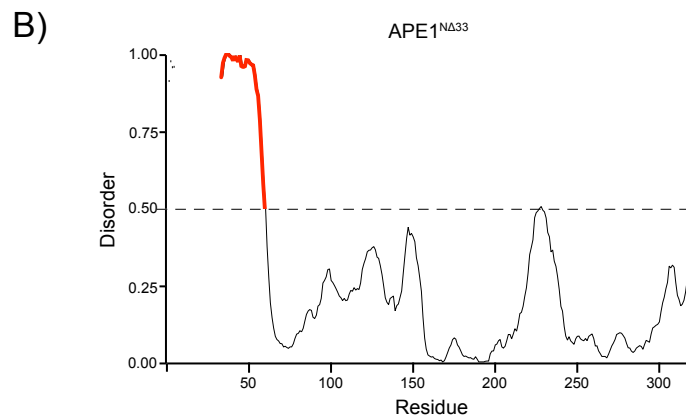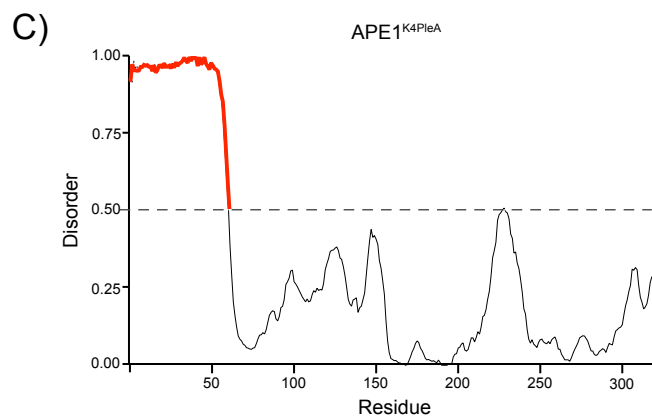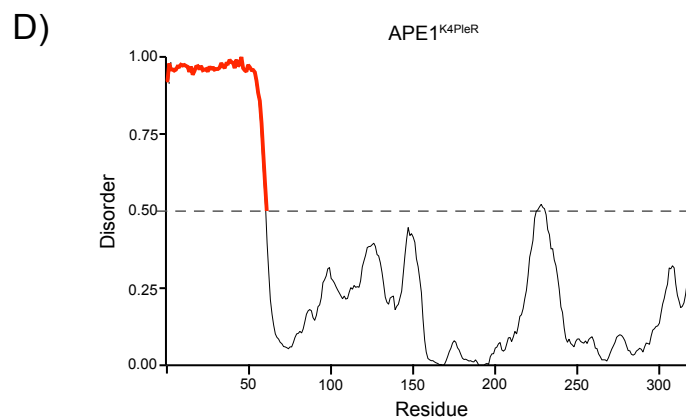

# Supp. Figure 4

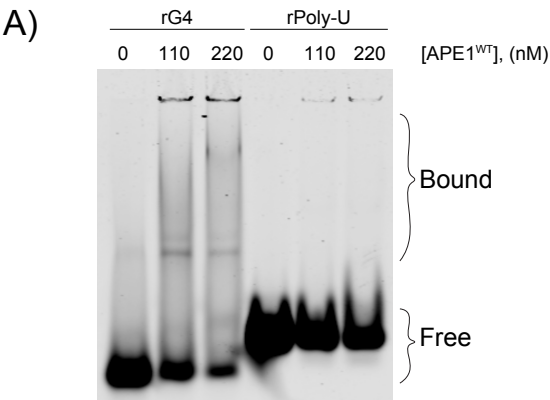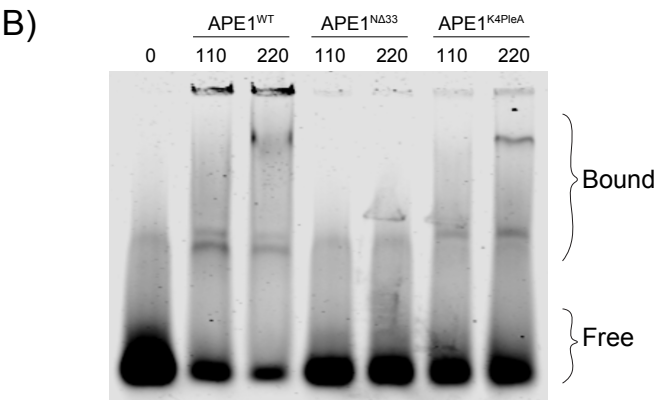

# Supp. Figure 5

A)

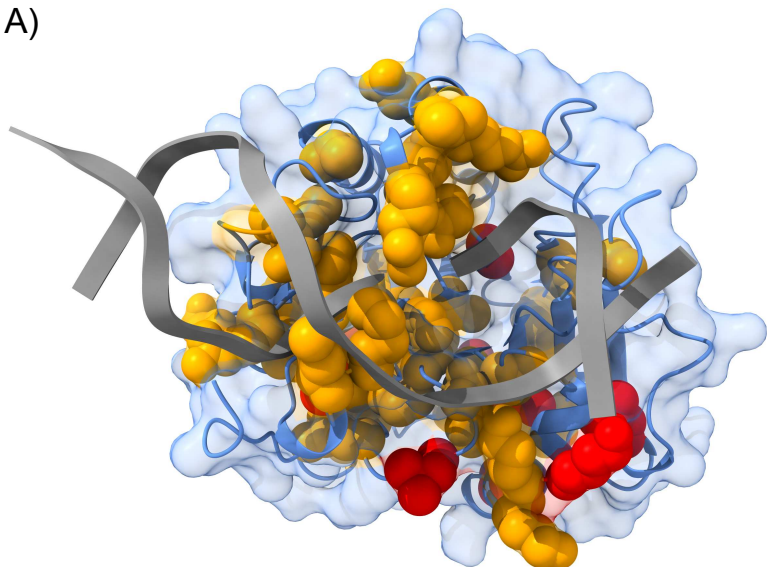

B)

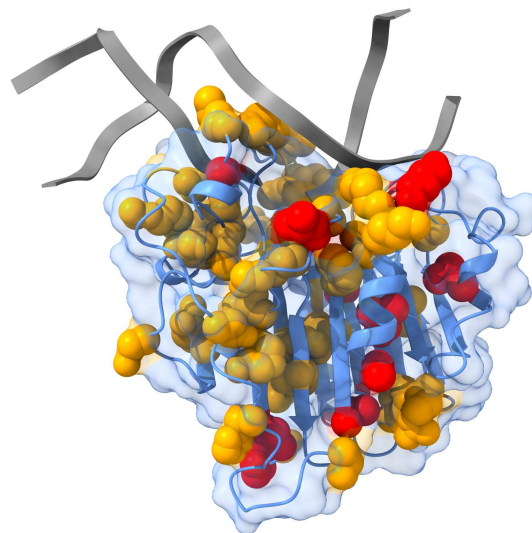

Supplement: gkaf168_Supplemental_Files [file gkaf168_supplemental_files.zip › Supplementary Figures_1-2-3-4-5_compressed.pdf]
